# Proteomic Analysis of Silk Viability in Maize Inbred Lines and Their Corresponding Hybrids

**DOI:** 10.1371/journal.pone.0144050

**Published:** 2015-12-02

**Authors:** Zhihui Ma, Yongtian Qin, Yafei Wang, Xiaofeng Zhao, Fangfang Zhang, Jihua Tang, Zhiyuan Fu

**Affiliations:** 1 Key Laboratory of Wheat and Maize Crops Science/Collaborative Innovation Center of Henan Grain Crops/College of Agronomy, Henan Agricultural University, Zhengzhou, 450002, China; 2 Hebi Academy of Agricultural Sciences, Hebi, 458030, China; Department of Agriculture and Food Western Australia, AUSTRALIA

## Abstract

A long period of silk viability is critical for a good seed setting rate in maize (*Zea mays* L.), especially for inbred lines and hybrids with a long interval between anthesis and silking. To explore the molecular mechanism of silk viability and its heterosis, three inbred lines with different silk viability characteristics (Xun928, Lx9801, and Zong3) and their two hybrids (Xun928×Zong3 and Lx9801×Zong3) were analyzed at different developmental stages by a proteomic method. The differentially accumulated proteins were identified by mass spectrometry and classified into metabolism, protein biosynthesis and folding, signal transduction and hormone homeostasis, stress and defense responses, and cellular processes. Proteins involved in nutrient (methionine) and energy (ATP) supply, which support the pollen tube growth in the silk, were important for silk viability and its heterosis. The additive and dominant effects at a single locus, as well as complex epistatic interactions at two or more loci in metabolic pathways, were the primary contributors for mid-parent heterosis of silk viability. Additionally, the proteins involved in the metabolism of anthocyanins, which indirectly negatively regulate local hormone accumulation, were also important for the mid-parent heterosis of silk viability. These results also might imply the developmental dependence of heterosis, because many of the differentially accumulated proteins made distinct contributions to the heterosis of silk viability at specific developmental stages.

## Introduction

The maize silk is functionally equivalent to the stigma and style of a typical pistil. It is a specialized elongated tissue that begins to senesce about 8–10 days after it emerges from the husks [1]. In normal conditions, pollination is completed within 1–2 days after silk emergence. Thus, a period of 8–10 days of silk viability is normally sufficient for seed setting. However, in hybrid seed production where plants are emasculated, a female parent with a long period of silk viability is critical for a good seed set. Thus, elucidating the molecular mechanisms of silk viability is necessary for the production of elite inbred lines and hybrid selection, which in turn contribute to maize hybrid seed production and field production.

Before losing viability, the maize silk can receive viable pollen, promotes pollen germination, guides pollen tube navigation, and aids pollen–ovule interactions [2,3]. Thus, silk viability has wide-ranging implications other than the specific function of supporting pollen germination through to fertilization. Under conducive conditions, compatible pollen grains hydrate and germinate pollen tubes. The tubes elongate via tip growth, penetrate the cell layers of the stigma, and enter the trichome, navigating within the transmitting tracts of the silk [4]. Usually, only a single pollen tube grows through the micropyle and eventually reaches the ovule where fertilization occurs [5]. All of these biological processes are critical for reproduction. Thus, several studies have analyzed the molecular mechanisms of pollen adhesion, pollen germination, pollen tube guidance, and pollen–ovule interactions [2, 3]. In pistil-interacting pollen tubes, genes involved in signal transduction, transcription, and pollen tube growth are highly expressed [6]. Cysteine-rich peptides (CRPs) for cell–cell communications were shown to be important for pollen–pistil interactions in several studies [7–10]. Genes involved in amino acid and lipid transport were shown to be important for, and unique to, reproductive processes in maize silks [11]. The cytosolic free Ca^2+^ concentration is another important factor in normal pollen tube growth and morphology [12]. A Ca^2+^ channel in pollen is formed by glutamate receptor-like proteins, which are regulated by D-serine in the pistil [13]. Nitric oxide, which has negative chemotropic activity in *lily* pollen tubes and is involved in pollen tube guidance in *Arabidopsis*, might function in tip-growth downstream of Ca^2+^ signaling [14]. For normal pollen tube growth, lipid transfer protein 5 (related to lily stigma cysteine-rich adhesin) and cysteine-rich receptor-like kinases were shown to be important for stigma-mediated reproductive processes in *Arabidopsis* [15] and maize [11], respectively. Normal pH and K^+^ homeostasis in the pollen tube are important for guidance of the pollen tube to the ovule [16]. cAMP was shown to play a second messenger role in regulating pollen tube growth and reorientation [17]. Receptor-like kinase proteins that maintain pollen tube integrity [18] and pollen tube attractants secreted by synergid cells [19] are also required to guide pollen tube growth to the embryo sacs to complete fertilization. A defensin-like cysteine-rich peptide protein encoded by *ZmES4* was shown to cause pollen tube burst in mature maize synergid cells by opening K^+^ channels [20].

Although many studies have identified key genes and molecules in the mechanisms of pollen–pistil interactions, pollen tube guidance, and pollen–ovule interactions in maize, few have focused on the molecular basis of silk viability, especially the genes and proteins related to silk viability before pollination. In practice, silks of maize hybrids always have much longer period time to accept pollen, complete double fertilization, and obtain seeds than their parental lines. Such as, silk viability of the improved lines of the local germplasm TangSPT that extensive used in maize breeding in China only has 5–6 days, while, 7–8 days silk viability were kept for their hybrid combinations with other lines from different heterotic group (S1 Table). However, the molecular mechanism of silk viability and its heterosis remain largely unstudied. The aims of this study were to: (1) identify key proteins related to silk viability at different silk developmental stages using a proteomics approach using three inbred lines, (2) illuminate the potential molecular mechanism of silk viability heterosis using two different hybrid combinations and its corresponding inbred lines, and (3) identify the common factors regulating both silk viability and its heterosis.

## Results

### Silk viability evaluated by seed setting rate

To evaluate differences in silk viability, the seed setting rate in the ear mid-base region (5–15 rounds from the ear base) was analyzed in three inbred lines and two hybrids (Table 1). At the five sampling stages, D_4_, D_6_, D_8_, D_10_, and D_12_, the average seed setting rate of three biological replications for the inbred lines Xun928 and Lx9801 was 99.3%, 97.4%, 95.0%, 26.0%, 13.5%, and 96.3%, 89.3%, 78.8%, 16.3%, 1.9%, respectively. The inbred line Zong3 sustained a high seed setting rate of 100% for all sampling stages. The ANOVA results showed that the difference of seed setting rate was significant between the parental inbred lines and their corresponding hybrids (*P* < 0.05), except for D_4_ between the inbred line Xun928 (*P* = 0.079), Zong3, and their hybrid combination Xun928×Zong3. The different sampling stages of Xun928, Lx9801, Xun928×Zong3, and Lx9801×Zong3 also showed significant differences (*P* < 0.01). Thus, least-significant difference (LSD) multiple comparisons were performed and showed that the seed setting rate between each sampling stage was significantly different both for Xun928 and Lx9801. However, non-significant difference was detected between D_4_ and D_6_ both for the two hybrids Xun928×Zong3 and Lx9801×Zong3.

**Table 1:** Analysis of variance for mid-base setting rate of inbred lines and corresponding hybrid combinations.

Sampling stage ^f^	Xun928	Lx9801	Zong3	Xun928×Zong3	Lx9801×Zong3	P-value ^h^	P-value ^i^
D_4_	99.3% ^a^	96.3% ^a^	100.0%	100.0% ^a^	100.0% ^a^	0.079	3.97E-4**
D_6_	97.4% ^b^	89.3% ^b^	100.0%	100.0% ^a^	99.6% ^a^	0.028*	1.91E-06**
D_8_	95.0% ^c^	78.8% ^c^	100.0%	95.6% ^b^	80.3% ^b^	7.32E-4**	9.00E-07**
D_10_	26.0% ^d^	16.3% ^d^	100.0%	84.6% ^c^	70.4% ^c^	2.84E-09**	1.81E-10**
D_12_	13.5% ^e^	1.9% ^e^	100.0%	80.2% ^d^	66.9% ^d^	3.09E-11**	3.6E-11**
P-value ^g^	4.10E-15**	7.10E-16**		1.25E-10**	1.52E-12**		

a, b, c, d, and e represent the results of LSD analysis at 0.05 significant level.

^f^: D_4_, D_6_, D_8_, D_10_, and D_12_ represents days after silk emerged above ligule of the husk outer leaf. Setting percentage was calculated by averaging three biological replications and each replication. Each replication consisted of 10 intact ears.

^g^: *P*-value is significance level among different sampling stages.

^h^: *P*-value is significance level between hybrids Xun928×Zong3 and its two parental lines at each sampling stage.

^i^: *P*-value is significance level between hybrids Lx9801×Zong3 and its two parental lines at each sampling stage.

*: Significant at 0.05 level.

**: Significant at 0.01 level.

The seed setting rate significantly decreased from D_8_ to D_10_ in both Xun928 and Lx9801 and the two hybrids. However, the decrease in the seed setting rate was slower in the hybrids than in the two inbred lines because of heterosis in the hybrids. Compared with those of the parental inbred lines, the seed setting rate of the hybrids fell between the mid-parent and high-parent values; i. e, seed setting rate showed partial dominant heterosis. Based on the phenotype of seed setting rate and heterostic degree, only the sampling stages with a significant difference at the 0.01 level were used for the proteomic analysis. Thus, the hybrids at D_8_, D_10_, and D_12_ were used in the proteomic analysis of heterosis, and the inbred lines Xun928, Lx9801, and Zong3 at stages D_6_, D_8_, D_10_, and D_12_ were used in the proteomic analysis of silk viability (Table 1).

### Differentially accumulated proteins

For the 2-DE analysis, only protein spots that showed the same trend in the three biological replicates were retrieved (S1 and S2 Figs). After normalization and ANOVA, only 3, 7, and 16 differentially accumulated protein spots were obtained for the inbred lines Xun928, Lx9801, and Zong3, respectively. These protein spots, which showed maximum changes more than 1.5-fold (*P* < 0.05) during the four sampling stages, were manually excised and analyzed by MS (Table 2 and S2 Table). Among the 26 differentially accumulated protein spots, 17 and 7 protein spots showed the lowest and the highest levels at D_6_, respectively (S3 Table). Meanwhile, 8 and 4 out of the 14 protein spots corresponding to D_6_ showed the highest and the lowest levels at D_12_, respectively. Among them, protein spot 46 gradually accumulated during silk development, while protein spot 61 gradually diminished.

**Table 2:** Differentially accumulated proteins identified by MS during silk development at four sampling stages in three inbred lines.

Spot No.^a^	Fold change^b^	Line name	Accession number^c^	Protein name^d^	Theoretical MW/PI	Protein Score^e^	Protein Score C. I.%^f^	Pep. Count	Gene name^g^	Gene position^h^	P-value
*Amino acid metabolism*											
8	4.1	Xun928	gi|414869037	putative methionine synthase family protein	84781.4/5.54	783	100	8	GRMZM2G149751	1:176865419–176870974	0
64	18.2	Zong3	gi|195636806	methylthioribose-1-phosphate isomerase [Zea mays]	38662.3/5.70	192	100	4	GRMZM2G139533	4:12797382–12792141	1.30E-148
*Biosynthesis of other secondary metabolites*											
16	2.1	Xun928	gi|195635735	GDSL-motif lipase/hydrolase-like protein [Zea mays]	41926.6/6.81	338	100	3	GRMZM2G700208	10:33361096–33356952	1.10E-39
53	6.5	Zong3	gi|1706374	Dihydroflavonol-4-reductase;	39172.9/5.48	1,060	100	11	GRMZM2G026930	3:216387831–216386092	9.70E-112
63	5.7	Zong3	gi|413944345	anthocyanidin 3-O-glucosyltransferase [Zea mays]	52096.3/5.83	592	100	11	GRMZM2G162755	6:120060819–120059072	3.80E-46
*Carbohydrate metabolism*											
3	3.1	Xun928	gi|414872668	alcohol dehydrogenase1 [Zea mays]	41572.9/6.15	162	100	2	GRMZM2G442658	1:274053872–274050420	4.00E-154
65	2.5	Zong3	gi|413917002	putative glyoxalase family protein [Zea mays]	32438.4/5.82	322	100	5	GRMZM2G181192	10:60096710–60090400	8.20E-115
*Stress and defense response*											
26	4.1	Lx9801	gi|12004294	T cytoplasm male sterility restorer factor 2 [Zea mays]	59750.9/6.69	369	100	2	GRMZM2G058675	9:34129896–34143610	2.90E-229
41	1.6	Zong3	gi|414873866	salt tolerance protein isoform 3 [Zea mays]	35309.2/4.92	221	100	4	GRMZM2G352415	1:297098531–297096881	1.50E-108
42	1.5	Zong3	gi|414873866	salt tolerance protein isoform 3 [Zea mays]	35309.2/4.92	377	100	6	GRMZM2G352415	1:297098531–297096881	1.50E-108
*Cell motility*											
48	2.2	Zong3	gi|414879552	putative actin family protein [Zea mays]	41920.9/5.18	691	100	7	GRMZM2G053284	3:175945092–175942615	7.50E-192
*Carbon fixation*											
40	2.9	Zong3	gi|414874045	RuBisCO large subunit-binding protein subunit	61418.8/5.20	557	100	7	GRMZM2G434173	1:300072472–300068298	9.10E-203
*Glycan biosynthesis and metabolism*											
56	3.8	Zong3	gi|12585309	Phosphoglucomutase, cytoplasmic 1;	63285.9/5.46	475	100	10	GRMZM2G109383	5:10871874–10866206	2.90E-261
62	2.7	Zong3	gi|12585310	Phosphoglucomutase, cytoplasmic 2;	63229.9/5.47	541	100	10	GRMZM2G023289	1:267953598–267959889	2.70E-267
*Nucleotide metabolism*											
46	3.1	Zong3	gi|414868742	adenine phosphoribosyl transferase 1 [Zea mays]	19507.4/5.14	321	100	5	GRMZM2G131907	1:164655824–164658422	8.60E-72
*Signal transduction*											
61	3.0	Zong3	gi|28373358	Chain A, Crystal Structure Of The Maize Zm-P60.1 Beta-Glucosidase	58185.4/5.44	280	100	9	GRMZM2G016890	10:34240659–34245626	1.20E-127
*Unknown*											
49	3.1	Zong3	gi|223975961	unknown [Zea mays]	34782.7/5.59	296	100	3	GRMZM2G175562	6:88908479–88909745	1.50E-136
19	2.8	Lx9801	gi|194689886	unknown [Zea mays]	17198.5/8.64	502	100	4	GRMZM2G152775	6:69732633–69729284	1.10E-41
37	3.7	Lx9801	gi|241920977	hypothetical protein SORBIDRAFT_01g020010	61322.1/5.67	373	100	9	GRMZM2G458208	5:31026372–31014140	8.10E-250
35	3.7	Lx9801	gi|257676173	unnamed protein product [Oryza sativa]	79272.0/7.19	430	100	3	GRMZM2G149751	1:176865419–176870974	2.1E-315
47	2.0	Zong3	gi|414884326	hypothetical protein ZEAMMB73_606346 [Zea mays]	31549.8/5.36	210	100	3	GRMZM2G051771	7:34010380–34006572	2.40E-122
66	3.2	Zong3	gi|219973780	unnamed protein product [Zea mays]	38084.1/6.00	760	100	11	GRMZM2G700188	7:108416493–108414610	2.30E-103
33	4.1	Lx9801	gi|224031021	unknown [Zea mays]	68688.9/5.36	1,230	100	18	GRMZM2G421857	4:235992822–235986865	2.60E-308
34	9.8	Lx9801	gi|194708072	unknown [Zea mays]	68547.2/5.46	236	100	8	GRMZM2G033208	9:22791995–22794986	1.10E-307
32	3.1	Lx9801	gi|293336560	hypothetical protein LOC100381658 [Zea mays]	60422.9/5.47	628	100	8	GRMZM2G003385	3:185895426–185890963	1.70E-256
43	2.1	Zong3	gi|257738098	unnamed protein product [Zea mays]	35967.1/5.35	973	100	10	GRMZM2G108153	2:25459872–25458525	3.40E-54

Notes

^a^ Spot No. corresponds to labels in 2-DE map.

^b^ Maximum fold changes between different developmental stages were calculated by ANOVA.

^c^ GenBank accession number of protein spot.

^d^ Protein name in NCBI database.

^e^ Protein scores were derived from ions scores as a non-probabilistic basis for ranking protein hits.

^f^ Confidence interval of the identified proteins.

^g^ Gene name retrieved from maize sequence (http://ensembl.gramene.org/Zea_mays/Info/Index) by cDNA blast.

^h^ Physical position determined by blast function in MaizeGDB.

For the heterosis analysis, 46, 47, and 37 protein spots with maximum changes of more than two-fold (*P* < 0.05) between the hybrid Xun928×Zong3 and its corresponding parents were retrieved at D_8_, D_10_, and D_12_, respectively (Table 3 and S2 Table). The corresponding numbers of protein spots with more than two-fold changes between the hybrid Lx9801×Zong3 and its two parents were 24, 37, and 24, respectively. Out of the 215 differentially accumulated proteins, about 57% (122 protein spots) were additively accumulated and 43% (93 protein spots) were non-additively accumulated in the two hybrids. Among the non-additively accumulated proteins, five interaction patterns were observed (Table 3); “−”, “− −”, “+”, “+ −”, and “+ +”, accounting for about 34% (32 protein spots), 3% (3 protein spots), 42% (39 protein spots), 12% (11 protein spots), and 9% (8 protein spots) of the non-additively accumulated proteins, respectively. The “− −” pattern was only detected in the hybrid Xun928×Zong3 at D_12_, and the “+ +” pattern was only detected at D_10_ and D_12_ in the two hybrids. The “+ −” pattern was found in the hybrid Xun928×Zong3 at D_8_ and D_12_ and the hybrid Lx9801×Zong3 at D_10_ and D_12_. The other two major non-additive accumulation patterns “+” and “−” were well distributed across the three sampling stages in each hybrid.

**Table 3:** Differentially accumulated proteins identified by MS between hybrids and corresponding inbred lines during different sampling stages.

Spot No.^a^	Fold change^b^	Heterotic pattern^c^	Hybrid name^d^	Accession number^e^	Protein name^f^	Theoretical MW/PI	Protein Score^g^	Protein Score C. I.% ^h^	Pep. Count		Gene name^i^	Gene position^j^	P-value
*Amino acid metabolism*													
270	2.2	A	XZ-D_12_	gi|195613424	N-acetyltransferase [Zea mays]	24346.3/5.95	208	100	7			9:152823282–152822299	1.40E-46
277	Y/N	+	LZ-D_12_	gi|195619648	IN2-1 protein [Zea mays]	27425.1/4.97	382	100	15	GRMZM2G162486		9:139006399–139008602	1.90E-76
80	Y/N	+	XZ-D_8_	gi|413923160	arginine decarboxylase isoform 1 [Zea mays]	71110.8/5.18	945	100	22	GRMZM2G374302		4:144866060–144868200	5.80E-137
121	Y/N	A	LZ-D_8_	gi|413933557	putative methionine synthase family protein isoform 1	84907.5/5.83	435	100	15	GRMZM2G112149		5:15569743–15564425	0.00E+00
148	Y/N	+	XZ-D_10_	gi|413950042	methionine adenosyltransferase [Zea mays]	46391.5/6.03	884	100	17	GRMZM2G117198		8:129153090–129156433	1.30E-189
194	2.9	A	LZ-D_10_	gi|413950043	methionine adenosyltransferase [Zea mays]	43389.9/5.57	577	100	15	GRMZM2G117198		8:129153090–129156433	1.30E-189
238	3.4	+ +	XZ-D_12_	gi|413950043	methionine adenosyltransferase [Zea mays]	43389.9/5.57	407	100	14	GRMZM2G117198		8:129153090–129156433	1.30E-189
155	Y/N	A	XZ-D_10_	gi|414869037	putative methionine synthase family protein	84781.4/5.54	472	100	19	GRMZM2G149751		1:176865419–176870974	0.00E+00
239	4.7	A	XZ-D_12_	gi|414869037	putative methionine synthase family protein	84781.4/5.54	466	100	19	GRMZM2G149751		1:176865419–176870974	0.00E+00
257	2.5	A	XZ-D_12_	gi|414886469	triosephosphate isomerase [Zea mays]	32685.8/6.14	555	100	15	GRMZM5G852968		7:143564086–143560301	6.60E-113
137	Y/N	+	LZ-D_8_	gi|50086699	betaine aldehyde dehydrogenase [Zea mays]	55739.3/5.34	262	100	10	GRMZM2G146754		4:79470324–79474910	4.40E-203
284	Y/N	A	LZ-D_12_	gi|50086699	betaine aldehyde dehydrogenase [Zea mays]	55739.3/5.34	347	100	13	GRMZM2G146754		4:79470324–79474910	4.40E-203
*Biosynthesis of other secondary metabolites*													
289	Y/N	-	LZ-D_12_	gi|195613588	tropinone reductase 2 [Zea mays]	28173.5/5.99	401	100	11	GRMZM2G152258		2:231084833–231086155	5.50E-61
163	16.5	A	XZ-D_10_	gi|195655583	O-methyltransferase ZRP4 [Zea mays]	40662.7/5.74	367	100	12	GRMZM2G385313		2:127725353–127723913	1.10E-46
171	Y/N	A	XZ-D_10_	gi|195655583	O-methyltransferase ZRP4 [Zea mays]	40662.7/5.74	413	100	14	GRMZM2G385313		2:127725353–127723913	1.10E-46
236	4.2	+ +	XZ-D_12_	gi|195655583	O-methyltransferase ZRP4 [Zea mays]	40662.7/5.74	271	100	10	GRMZM2G385313		2:127725353–127723913	1.10E-46
243	6.1	+	XZ-D_12_	gi|195655583	O-methyltransferase ZRP4 [Zea mays]	40662.7/5.74	358	100	15	GRMZM2G385313		2:127725353–127723913	1.10E-46
89	Y/N	+ -	XZ-D_8_	gi|413920184	O-methyltransferase ZRP4 [Zea mays]	39557.4/5.52	509	100	8	GRMZM2G349791		4:1300771–1299504	1.00E-43
118	8.4	-	LZ-D_8_	gi|413920184	O-methyltransferase ZRP4 [Zea mays]	39557.4/5.52	672	100	14	GRMZM2G349791		4:1300771–1299504	1.00E-43
150	Y/N	A	XZ-D_10_	gi|413920184	O-methyltransferase ZRP4 [Zea mays]	39557.4/5.52	797	100	16	GRMZM2G349791		4:1300771–1299504	1.00E-43
272	Y/N	+	XZ-D_12_	gi|413920184	O-methyltransferase ZRP4 [Zea mays]	39557.4/5.52	430	100	13	GRMZM2G349791		4:1300771–1299504	1.00E-43
291	3.9	A	LZ-D_12_	gi|413920184	O-methyltransferase ZRP4 [Zea mays]	39557.4/5.52	662	100	15	GRMZM2G349791		4:1300771–1299504	1.00E-43
79	Y/N	A	XZ-D_8_	gi|104303692	UDP-glucose flavonoid-3-O-glucosyltransferase [Zea mays]	49229.9/5.33	593	100	16	GRMZM2G165390		9:11781405–11779704	1.20E-60
197	Y/N	-	LZ-D_10_	gi|162463323	anthocyanidin 3-O-glucosyltransferase [Zea mays]	49251.8/5.39	105	100	9	GRMZM2G165390		9:11781402–11779647	1.80E-62
99	Y/N	A	XZ-D_8_	gi|413944345	anthocyanidin 3-O-glucosyltransferase [Zea mays]	52096.3/5.83	934	100	21	GRMZM2G162755		6:120060819–120059072	3.80E-46
117	Y/N	+	LZ-D_8_	gi|413944345	anthocyanidin 3-O-glucosyltransferase [Zea mays]	52096.3/5.83	140	100	9	GRMZM2G162755		6:120060819–120059072	3.80E-46
122	Y/N	A	LZ-D_8_	gi|413944345	anthocyanidin 3-O-glucosyltransferase [Zea mays]	52096.3/5.83	411	100	17	GRMZM2G162755		6:120060819–120059072	3.80E-46
123	6.8	A	LZ-D_8_	gi|413944345	anthocyanidin 3-O-glucosyltransferase [Zea mays]	52096.3/5.83	560	100	20	GRMZM2G162755		6:120060819–120059072	3.80E-46
159	4.8	A	XZ-D_10_	gi|413944345	anthocyanidin 3-O-glucosyltransferase [Zea mays]	52096.3/5.83	419	100	19	GRMZM2G162755		6:120060819–120059072	3.80E-46
200	Y/N	A	LZ-D_10_	gi|413944345	anthocyanidin 3-O-glucosyltransferase [Zea mays]	52096.3/5.83	457	100	19	GRMZM2G162755		6:120060819–120059072	3.80E-46
201	6.3	A	LZ-D_10_	gi|413944345	anthocyanidin 3-O-glucosyltransferase [Zea mays]	52096.3/5.83	668	100	20	GRMZM2G162755		6:120060819–120059072	3.80E-46
212	10.1	+ +	LZ-D_10_	gi|413944345	anthocyanidin 3-O-glucosyltransferase [Zea mays]	52096.3/5.83	518	100	16	GRMZM2G162755		6:120060819–120059072	3.80E-46
241	Y/N	A	XZ-D_12_	gi|413944345	anthocyanidin 3-O-glucosyltransferase [Zea mays]	52096.3/5.83	519	100	17	GRMZM2G162755		6:120060819–120059072	3.80E-46
119	Y/N	A	LZ-D_8_	gi|413944348	anthocyanidin 3-O-glucosyltransferase [Zea mays]	52266.8/5.36	517	100	17	GRMZM2G383404		6:120201779–120203533	6.90E-47
152	Y/N	A	XZ-D_10_	gi|413944348	anthocyanidin 3-O-glucosyltransferase [Zea mays]	52266.8/5.36	653	100	16	GRMZM2G383404		6:120201779–120203533	6.90E-47
231	Y/N	A	LZ-D_10_	gi|413944348	anthocyanidin 3-O-glucosyltransferase [Zea mays]	52266.8/5.36	393	100	15	GRMZM2G383404		6:120201779–120203533	6.90E-47
202	5.3	A	LZ-D_10_	gi|414590349	anthocyanidin 3-O-glucosyltransferase [Zea mays]	47063.1/5.84	626	100	17	GRMZM2G180283		2:202537696–202536156	9.90E-60
76	9.2	A	XZ-D_8_	gi|414881303	anthocyaninless1 [Zea mays]	39100.8/5.48	1,110	100	20	GRMZM2G026930		3:216387830–216386070	4.20E-111
138	12.9	A	LZ-D_8_	gi|414881303	anthocyaninless1 [Zea mays]	39100.8/5.48	957	100	15	GRMZM2G026930		3:216387830–216386070	4.20E-111
188	18.8	A	XZ-D_10_	gi|414881303	anthocyaninless1 [Zea mays]	39100.8/5.48	917	100	18	GRMZM2G026930		3:216387830–216386070	4.20E-111
224	15.1	+	LZ-D_10_	gi|414881303	anthocyaninless1 [Zea mays]	39100.8/5.48	966	100	20	GRMZM2G026930		3:216387830–216386070	4.20E-111
285	Y/N	A	LZ-D_12_	gi|414881303	anthocyaninless1 [Zea mays]	39100.8/5.48	857	100	17	GRMZM2G026930		3:216387830–216386070	4.20E-111
96	3.6	-	XZ-D_8_	gi|195635735	GDSL-motif lipase/hydrolase-like protein [Zea mays]	41926.6/6.81	76	99.614	5	GRMZM2G700208		10:33361036–33356952	1.10E-39
255	Y/N	-	XZ-D_12_	gi|413920639	chitinase 1 [Zea mays]	31165.5/4.97	116	100	5	GRMZM2G358153		4:12097106–12098325	0.00017
275	Y/N	+	LZ-D_12_	gi|413920639	chitinase 1 [Zea mays]	31165.5/4.97	80	99.807	4	GRMZM2G358153		4:12097106–12098325	0.00017
127	Y/N	+	LZ-D_8_	gi|414878829	glutathione S-transferase4 [Zea mays]	23879.7/5.96	378	100	8	GRMZM2G034083		3:128543799–128546078	9.70E-199
216	Y/N	+ -	LZ-D_10_	gi|414878829	glutathione S-transferase4 [Zea mays]	23879.7/5.96	369	100	8	GRMZM2G034083		3:128543799–128546078	9.70E-199
298	Y/N	A	LZ-D_12_	gi|414878829	glutathione S-transferase4 [Zea mays]	23879.7/5.96	314	100	6	GRMZM2G034083		3:128543799–128546078	9.70E-199
217	Y/N	+	LZ-D_10_	gi|162460516	Glutathione transferase III(b) [Zea mays]	23865.6/5.96	267	100	5	GRMZM2G146246		3:155017244–155015982	1.00E-52
*Carbohydrate metabolism*													
223	Y/N	A	LZ-D_10_	gi|20385155	NADPH-dependent reductase [Zea mays]	39172.9/5.48	739	100	19	GRMZM2G026930		3:216387831–216386092	9.70E-112
74	Y/N	A	XZ-D_8_	gi|195643366	APx2—Cytosolic Ascorbate Peroxidase [Zea mays]	27298.8/5.28	890	100	13	GRMZM2G140667		2:219260991–219258343	1.10E-96
140	Y/N	A	LZ-D_8_	gi|195643366	APx2—Cytosolic Ascorbate Peroxidase [Zea mays]	27298.8/5.28	629	100	13	GRMZM2G140667		2:219260991–219258343	1.10E-96
190	Y/N	A	XZ-D_10_	gi|195643366	APx2—Cytosolic Ascorbate Peroxidase [Zea mays]	27298.8/5.28	762	100	13	GRMZM2G140667		2:219260991–219258343	1.10E-96
268	Y/N	A	XZ-D_12_	gi|195643366	APx2—Cytosolic Ascorbate Peroxidase [Zea mays]	27298.8/5.28	684	100	15	GRMZM2G140667		2:219260991–219258343	1.10E-96
287	Y/N	A	LZ-D_12_	gi|195643366	APx2—Cytosolic Ascorbate Peroxidase [Zea mays]	27298.8/5.28	766	100	13	GRMZM2G140667		2:219260991–219258343	1.10E-96
104	Y/N	A	XZ-D_8_	gi|413942605	6-phosphogluconate dehydrogenase isoenzyme B	53102.9/5.93	638	100	24	GRMZM2G127798		6:57906012–57903707	3.00E-243
176	Y/N	A	XZ-D_10_	gi|413942605	6-phosphogluconate dehydrogenase isoenzyme B	53102.9/5.93	460	100	18	GRMZM2G127798		6:57906012–57903707	3.00E-243
211	Y/N	+ +	LZ-D_10_	gi|413942605	6-phosphogluconate dehydrogenase isoenzyme B	53102.9/5.93	401	100	14	GRMZM2G127798		6:57906012–57903707	3.00E-243
247	Y/N	A	XZ-D_12_	gi|413942605	6-phosphogluconate dehydrogenase isoenzyme B	53102.9/5.93	408	100	15	GRMZM2G127798		6:57906012–57903707	3.00E-243
177	Y/N	A	XZ-D_10_	gi|413944222	ppi-phosphofructokinase [Zea mays]	61640.1/6.3	291	100	18	GRMZM2G059151		6:115378780–115372697	2.50E-239
232	Y/N	+	LZ-D_10_	gi|413949561	sucrose-phosphatase1 [Zea mays]	47669.2/5.8	255	100	10	GRMZM2G055489		8:115837557–115832290	6.90E-134
173	Y/N	A	XZ-D_10_	gi|414865246	putative alcohol dehydrogenase superfamily	41850.2/6.1	551	100	14	GRMZM2G154007		1:19329011–19326354	4.80E-110
95	Y/N	A	XZ-D_8_	gi|414866238	APx1-Cytosolic Ascorbate Peroxidase [Zea mays]	27467.9/5.64	393	100	11	GRMZM2G137839		1:43682927–43685858	3.20E-113
149	4.0	+	XZ-D_10_	gi|414868220	putative enolase family protein isoform 1 [Zea mays]	48369.6/5.59	743	100	19	GRMZM2G048371		1:125087719–125081660	4.40E-210
105	Y/N	A	XZ-D_8_	gi|414871407	GDP-mannose 3,5-epimerase 1 [Zea mays]	43337.3/5.99	405	100	17	GRMZM2G124434		1:240922252–240925270	3.80E-188
160	5.1	A	XZ-D_10_	gi|414872918	UDP-glucose 6-dehydrogenase [Zea mays]	56749.2/6.23	648	100	19	GRMZM2G328500		1:278196990–278199108	4.20E-237
165	5.2	A	XZ-D_10_	gi|414872919	UDP-glucose 6-dehydrogenase isoform 1 [Zea mays]	53495.4/5.71	407	100	17	GRMZM2G328500		1:278196990–278199108	4.20E-237
204	Y/N	+	LZ-D_10_	gi|414878099	ATP-citrate synthase [Zea mays]	46948.1/5.58	380	100	16	GRMZM2G034083		3:128543602–128547481	9.70E-199
292	Y/N	+	LZ-D_12_	gi|414878099	ATP-citrate synthase [Zea mays]	46948.1/5.58	354	100	16	GRMZM2G034083		3:128543602–128547481	9.70E-199
86	Y/N	A	XZ-D_8_	gi|75172084	Sucrose-phosphatase 1;	47583/5.48	634	100	18	GRMZM2G055489		8:115838485–115832372	2.60E-141
120	Y/N	A	LZ-D_8_	gi|75172084	Sucrose-phosphatase 1;	47583/5.48	436	100	17	GRMZM2G055489		8:115838485–115832372	2.60E-141
151	Y/N	A	XZ-D_10_	gi|75172084	Sucrose-phosphatase 1;	47583/5.48	466	100	16	GRMZM2G055489		8:115838485–115832372	2.60E-141
*Cell structure*													
220	3.8	A	LZ-D_10_	gi|8928413	Tubulin beta-4 chain	50784.1/4.86	771	100	21	GRMZM2G066191		5:14874004–14870370	1.90E-209
262	4.5	A	XZ-D_12_	gi|8928413	Tubulin beta-4 chain	50784.1/4.86	561	100	20	GRMZM2G066191		5:14874004–14870370	1.90E-209
283	Y/N	+ -	LZ-D_12_	gi|8928413	Tubulin beta-4 chain	50784.1/4.86	675	100	21	GRMZM2G066191		5:14874004–14870370	1.90E-209
*Stress and defense response*													
164	Y/N	+	XZ-D_10_	gi|162459030	legumin-like protein [Zea mays]	38046.6/5.79	611	100	12	GRMZM5G801289		8:132711366–132712942	3.30E-118
207	Y/N	+	LZ-D_10_	gi|162459030	legumin-like protein [Zea mays]	38046.6/5.79	494	100	12	GRMZM5G801289		8:132711366–132712942	3.30E-118
246	5.0	A	XZ-D_12_	gi|162459030	legumin-like protein [Zea mays]	38046.6/5.79	653	100	11	GRMZM5G801289		8:132711366–132712942	3.30E-118
98	Y/N	A	XZ-D_8_	gi|195629806	legumin-like protein [Zea mays]	38076.6/5.79	872	100	12	GRMZM2G005552		8:132711342–132712943	4.20E-118
244	Y/N	A	XZ-D_12_	gi|195629806	legumin-like protein [Zea mays]	38076.6/5.79	750	100	13	GRMZM2G005552		8:132711342–132712943	4.20E-118
111	4.8	+ -	XZ-D_8_	gi|413934538	peroxidase 39 isoform 2 [Zea mays]	35814.3/7.59	654	100	14	GRMZM2G085967		5:47608191–47611493	8.40E-97
115	Y/N	A	XZ-D_8_	gi|413934538	peroxidase 39 isoform 2 [Zea mays]	35814.3/7.59	663	100	15	GRMZM2G085967		5:47608191–47611493	8.40E-97
213	Y/N	A	LZ-D_10_	gi|413934538	peroxidase 39 isoform 2 [Zea mays]	35814.3/7.59	378	100	13	GRMZM2G085967		5:47608191–47611493	8.40E-97
214	Y/N	A	LZ-D_10_	gi|413934538	peroxidase 39 isoform 2 [Zea mays]	35814.3/7.59	459	100	17	GRMZM2G085967		5:47608191–47611493	8.40E-97
248	3.6	-	XZ-D_12_	gi|413934538	peroxidase 39 isoform 2 [Zea mays]	35814.3/7.59	516	100	13	GRMZM2G085967		5:47608191–47611493	8.40E-97
183	Y/N	A	XZ-D_10_	gi|75994013	pathogenesis-related protein 5 [Zea mays subsp. mays]	18061.3/4.87	216	100	2	GRMZM2G402631		1:178036019–178035437	4.50E-38
110	Y/N	A	XZ-D_8_	gi|414870957	secretory protein [Zea mays]	24524.1/4.82	242	100	6	GRMZM2G153208		1:228705641–228704654	1.70E-66
147	Y/N	-	XZ-D_10_	gi|414870957	secretory protein [Zea mays]	24524.1/4.82	333	100	7	GRMZM2G153208		1:228705641–228704654	1.70E-66
*Energy metabolism*													
87	Y/N	A	XZ-D_8_	gi|28172915	cytosolic 3-phosphoglycerate kinase [Zea mays]	31662.8/5.01	850	100	16	GRMZM2G382914		5:84861898–84858488	1.70E-128
235	Y/N	A	XZ-D_12_	gi|28172915	cytosolic 3-phosphoglycerate kinase [Zea mays]	31662.8/5.01	494	100	18	GRMZM2G382914		5:84861898–84858488	1.70E-128
88	Y/N	A	XZ-D_8_	gi|413935733	phosphoglycerate kinase isoform 3 [Zea mays]	42469.8/5.65	988	100	23	GRMZM2G382914		5:84858478–84861909	8.60E-175
237	Y/N	+	XZ-D_12_	gi|413935733	phosphoglycerate kinase isoform 3 [Zea mays]	42469.8/5.65	839	100	26	GRMZM2G382914		5:84858478–84861909	8.60E-175
199	5.8	-	LZ-D_10_	gi|11467189	ATP synthase CF1 alpha subunit [Zea mays]	55729.4/5.87	692	100	28	GRMZM2G385622		2:200755769–200756023	2.90E-245
145	2.8	-	XZ-D_10_	gi|162464321	malate dehydrogenase, cytoplasmic [Zea mays]	35909.3/5.77	574	100	16	GRMZM2G415359		1:231403594–231398465	1.40E-156
182	2.2	-	XZ-D_10_	gi|195612184	ATP synthase delta chain [Zea mays]	21269.3/5.72	143	100	6	GRMZM2G171628		2:202325467–202328755	1.60E-70
71	Y/N	A	XZ-D_8_	gi|195640660	formate dehydrogenase 1 [Zea mays]	41678.2/6.32	455	100	13	GRMZM2G049811		9:49450491–49446497	1.10E-156
124	Y/N	-	LZ-D_8_	gi|414589713	putative oxidoreductase, aldo/keto reductase	36998/6.07	457	100	11	GRMZM2G087507		2:188279947–188276596	1.70E-84
*Carbon fixation*													
67	3.0	A	XZ-D_8_	gi|11467200	ribulose-1,5-bisphosphate carboxylase/oxygenase large subunit	53294.6/6.33	842	100	27	GRMZM2G469277;GRMZM2G062854		6:160979914–160980168	3.10E-241
106	Y/N	+	XZ-D_8_	gi|11467200	ribulose-1,5-bisphosphate carboxylase/oxygenase large subunit	53294.6/6.33	575	100	18	GRMZM2G469277;GRMZM2G062854		6:160979914–160980168	3.10E-241
178	7.4	A	XZ-D_10_	gi|11467200	ribulose-1,5-bisphosphate carboxylase/oxygenase large subunit	53294.6/6.33	577	100	20	GRMZM2G469277;GRMZM2G062854		6:160979914–160980168	3.10E-241
*Protein biosynthesis and folding*													
91	2.7	A	XZ-D_8_	gi|413922095	retrotransposon protein Ty1-copia subclass [Zea mays]	28923.7/6.31	253	100	8	GRMZM2G073079		4:70093844–70091439	6.10E-39
94	4.3	A	XZ-D_8_	gi|413919640	glycine-rich protein 2b [Zea mays]	20576.1/5.92	469	100	10	GRMZM5G895313		10:143487212–143487775	2.40E-35
161	Y/N	A	XZ-D_10_	gi|413932420	putative translation elongation/initiation factor family	46309.1/6.85	244	100	14	GRMZM2G313678		5:1033629–1037919	7.50E-176
72	Y/N	A	XZ-D_8_	gi|162464130	eukaryotic translation initiation factor 5A [Zea mays]	17713.8/5.61	536	100	10	GRMZM2G144030		7:163045095–163042293	1.30E-70
146	Y/N	A	XZ-D_10_	gi|162464130	eukaryotic translation initiation factor 5A [Zea mays]	17713.8/5.61	205	100	10	GRMZM2G144030		7:163045095–163042293	1.30E-70
112	3.4	A	XZ-D_8_	gi|414887577	elongation factor 1-delta 1 [Zea mays]	24951.4/4.39	392	100	10	GRMZM2G031545		7:165426108–165423624	1.40E-64
251	Y/N	A	XZ-D_12_	gi|414887577	elongation factor 1-delta 1 [Zea mays]	24951.4/4.39	148	100	7	GRMZM2G031545		7:165426108–165423624	1.40E-64
70	Y/N	A	XZ-D_8_	gi|414589578	chaperonin isoform 1 [Zea mays]	25558.7/8.67	465	100	11	GRMZM2G399284		2:184583814–184578857	1.20E-67
258	Y/N	A	XZ-D_12_	gi|414589578	chaperonin isoform 1 [Zea mays]	25558.7/8.67	220	100	8	GRMZM2G399284		2:184583814–184578857	1.20E-67
260	Y/N	A	XZ-D_12_	gi|414589578	chaperonin isoform 1 [Zea mays]	25558.7/8.67	213	100	13	GRMZM2G399284		2:184583814–184578857	1.20E-67
279	Y/N	A	LZ-D_12_	gi|414589578	chaperonin isoform 1 [Zea mays]	25558.7/8.67	291	100	13	GRMZM2G399284		2:184583814–184578857	1.20E-67
252	3.4	A	XZ-D_12_	gi|145666464	protein disulfide isomerase [Zea mays]	56921/5.01	783	100	31	GRMZM2G393320		4:14881672–14877150	5.30E-214
229	Y/N	A	LZ-D_10_	gi|413933376	proteasome subunit beta type [Zea mays]	23240/5.6	95	99.994	10	GRMZM2G111566		5:12576599–12579751	5.20E-81
288	Y/N	-	LZ-D_12_	gi|413933376	proteasome subunit beta type [Zea mays]	23240/5.6	224	100	10	GRMZM2G111566		5:12576599–12579751	5.20E-81
*Glycan biosynthesis and metabolism*													
267	Y/N	- -	XZ-D_12_	gi|195629642	lichenase-2 precursor [Zea mays]	35062.1/5.68	72	98.78	6	GRMZM2G137535		6:142506341–142502273	5.70E-75
290	Y/N	+ +	LZ-D_12_	gi|195629642	lichenase-2 precursor [Zea mays]	35062.1/5.68	234	100	5	GRMZM2G137535		6:142506341–142502273	5.70E-75
90	3.8	A	XZ-D_8_	gi|34588146	Alpha-1,4-glucan-protein synthase	41690.8/5.75	715	100	21	GRMZM2G073725		1:248925143–248922623	1.20E-186
*Lipid metabolism*													
198	Y/N	-	LZ-D_10_	gi|12620877	lipoxygenase [Zea mays]	96587.2/5.71	419	100	28	GRMZM2G109056		1:264266453–264290362	0.00E+00
281	Y/N	+ -	LZ-D_12_	gi|413933924	epoxide hydrolase 2 isoform 1 [Zea mays]	35469.8/5.09	118	100	7	GRMZM2G032910		5:24638574–24636922	5.20E-49
*Nucleotide metabolism*													
225	Y/N	+	LZ-D_10_	gi|414589043	pyrimidine-specific ribonucleoside hydrolase rihB	34329.6/5.36	254	100	5	GRMZM2G104999		2:166013604–166017041	1.60E-116
266	Y/N	+ -	XZ-D_12_	gi|414589043	pyrimidine-specific ribonucleoside hydrolase rihB	34329.6/5.36	161	100	4	GRMZM2G104999		2:166013604–166017041	1.60E-116
286	Y/N	+	LZ-D_12_	gi|414589043	pyrimidine-specific ribonucleoside hydrolase rihB	34329.6/5.36	260	100	7	GRMZM2G104999		2:166013604–166017041	1.60E-116
*Plant hormone biosynthesis and signal transduction*													
107	2.9	A	XZ-D_8_	gi|195645942	cytokinin-O-glucosyltransferase 2 [Zea mays]	53489.2/5.86	286	100	11	GRMZM2G083130		3:135786100–135784404	1.10E-94
274	Y/N	+ -	LZ-D_12_	gi|413924376	acc oxidase [Zea mays]	35699.8/4.97	217	100	12	GRMZM2G126732		4:177660129–177657861	1.60E-109
168	4.0	A	XZ-D_10_	gi|414867333	allene oxide cyclase 4 [Zea mays]	25932.4/9.05	140	100	8	GRMZM2G077316		1:76741280–76740459	3.50E-59
299	Y/N	-	LZ-D_12_	gi|195652523	ABA-responsive protein [Zea mays]	29250.4/6.14	263	100	8	GRMZM2G106622		10:14307592–14310405	5.10E-74
193	2.4	-	XZ-D_10_	gi|21730839	Chain C, Crystal Structure Of Auxin-Binding Protein 1 In Complex With 1-Naphthalene Acetic Acid	18552.2/5.24	157	100	7	GRMZM2G116204		3:133916933–133921384	6.00E-87
263	Y/N	-	XZ-D_12_	gi|413925162	putative O-Glycosyl hydrolase superfamily protein [Zea mays]	83731.8/5.78	294	100	15			7:144964081–144966447	2.60E-221
69	Y/N	A	XZ-D_8_	gi|413939100	pyrophosphate-energized proton pump1, partial [Zea mays]	16441.6/9.6	90	99.981	3	GRMZM2G090718		5:210647780–210643103	3.60E-41
254	Y/N	A	XZ-D_12_	gi|413944231	gibberellin receptor GID1L2 [Zea mays]	34820.6/5	463	100	12	GRMZM2G156310		6:115727511–115728876	2.80E-57
*Unknown*													
187	2.8	+	XZ-D_10_	gi|414589057	hypothetical protein ZEAMMB73_276484 [Zea	46817.3/5.45	645	100	20	GRMZM2G084881		2:166253571–166248891	1.80E-170
265	11.2	A	XZ-D_12_	gi|414589057	hypothetical protein ZEAMMB73_276484 [Zea	46817.3/5.45	543	100	20	GRMZM2G084881		2:166253571–166248891	1.80E-170
109	Y/N	A	XZ-D_8_	gi|414870644	hypothetical protein ZEAMMB73_435161 [Zea mays]	14648.4/5	158	100	5	GRMZM2G078022		1:220950014–220945296	4.80E-31
126	Y/N	A	LZ-D_8_	gi|414584728	hypothetical protein ZEAMMB73_474117 [Zea mays]	28283.2/5.83	269	100	9	GRMZM2G165535		2:964185–961339	1.70E-105
128	5.4	A	LZ-D_8_	gi|414876731	hypothetical protein ZEAMMB73_561858 [Zea mays]	33565.8/5.96	521	100	13	GRMZM2G120304		3: 31902191–31898927	8.30E-51
169	4.6	+	XZ-D_10_	gi|414876731	hypothetical protein ZEAMMB73_561858 [Zea mays]	33565.8/5.96	297	100	9	GRMZM2G120304		3: 31902191–31898927	8.30E-51
113	Y/N	A	XZ-D_8_	gi|414587271	hypothetical protein ZEAMMB73_690514 [Zea mays]	27760.2/5.07	542	100	16	GRMZM5G806182		2:54474692–54473385	
114	Y/N	A	XZ-D_8_	gi|414587271	hypothetical protein ZEAMMB73_690514 [Zea mays]	27760.2/5.07	425	100	11	GRMZM5G806182		2:54474692–54473385	
135	Y/N	A	LZ-D_8_	gi|414587271	hypothetical protein ZEAMMB73_690514 [Zea mays]	27760.2/5.07	699	100	15	GRMZM5G806182		2:54474692–54473385	
179	Y/N	+	XZ-D_10_	gi|414587271	hypothetical protein ZEAMMB73_690514 [Zea mays]	27760.2/5.07	132	100	8	GRMZM5G806182		2:54474692–54473385	
180	Y/N	A	XZ-D_10_	gi|414587271	hypothetical protein ZEAMMB73_690514 [Zea mays]	27760.2/5.07	496	100	14	GRMZM5G806182		2:54474692–54473385	
245	Y/N	A	XZ-D_12_	gi|414588073	hypothetical protein ZEAMMB73_739939 [Zea mays]	38290.9/8.95	202	100	6	GRMZM2G330377		2:73644958–73649833	1.50E-19
108	4.0	A	XZ-D_8_	gi|414587991	hypothetical protein ZEAMMB73_919014 [Zea mays]	42290.4/6.53	524	100	14	GRMZM2G050131		2:102982712–102984376	0.014
81	Y/N	A	XZ-D_8_	gi|414879972	hypothetical protein ZEAMMB73_969630 [Zea mays]	60422.9/5.47	990	100	21	GRMZM2G003385		3:185895426–185890963	1.70E-256
233	Y/N	A	XZ-D_12_	gi|195642378	hypothetical protein [Zea mays]	42563.1/5.51	209	100	12	GRMZM2G072909		9:65142126–65155480	9.30E-130
189	Y/N	-	XZ-D_10_	gi|195640116	hypothetical protein [Zea mays]	27609.9/5.12	292	100	9	GRMZM2G430600		7:174328795–174326866	9.90E-55
116	Y/N	A	LZ-D_8_	gi|195620226	hypothetical protein [Zea mays]	17568/5.51	514	100	10	GRMZM2G041258		6:106123618–106122830	6.40E-28
228	Y/N	A	LZ-D_10_	gi|195640026	hypothetical protein [Zea mays]	17528.9/5.35	150	100	9	GRMZM2G041258		6:106123618–106122833	3.90E-28
210	5.4	+ +	LZ-D_10_	gi|413950795	hypothetical protein ZEAMMB73_038317 [Zea mays]	46509.6/6.11	478	100	21	GRMZM2G432128		8:147229052–147224985	1.30E-196
219	Y/N	A	LZ-D_10_	gi|413944150	hypothetical protein ZEAMMB73_357615 [Zea mays]	35990.9/5.03	970	100	14	GRMZM2G701082		6:113741028–113738663	4.50E-15
221	Y/N	A	LZ-D_10_	gi|413944150	hypothetical protein ZEAMMB73_357615 [Zea mays]	35990.9/5.03	546	100	16	GRMZM2G701082		6:113741028–113738663	4.50E-15
282	Y/N	A	LZ-D_12_	gi|413944150	hypothetical protein ZEAMMB73_357615 [Zea mays]	35990.9/5.03	830	100	13	GRMZM2G701082		6:113741028–113738663	4.50E-15
157	7.3	A	XZ-D_10_	gi|413921122	hypothetical protein ZEAMMB73_482448 [Zea mays]	87111.7/5.78	430	100	23			4:28145897–28146077	0
174	Y/N	A	XZ-D_10_	gi|413939433	hypothetical protein ZEAMMB73_631326 [Zea mays]	53487.6/6	284	100	15	GRMZM5G806449		5:215337548–215346511	6.10E-197
259	Y/N	A	XZ-D_12_	gi|413948610	hypothetical protein ZEAMMB73_645738 [Zea mays]	18144.2/5.3	156	100	4	GRMZM2G045664		8:74457615–74456935	2.30E-46
278	Y/N	+	LZ-D_12_	gi|413948610	hypothetical protein ZEAMMB73_645738 [Zea mays]	18144.2/5.3	206	100	4	GRMZM2G045664		8:74457615–74456935	2.30E-46
166	3.8	+ +	XZ-D_10_	gi|413952229	hypothetical protein ZEAMMB73_660929 [Zea mays]	12646.2/7.9	179	100	4	GRMZM2G159643		8:171408802–171405316	1.10E-48
186	Y/N	A	XZ-D_10_	gi|413935382	hypothetical protein ZEAMMB73_705624 [Zea mays]	55418.5/6.53	465	100	16	GRMZM2G370852		5:72953434–72949542	5.70E-217
218	3.5	-	LZ-D_10_	gi|413949747	hypothetical protein ZEAMMB73_945417 [Zea mays]	49720.5/6.99	602	100	21	GRMZM2G083016		8:120301530–120303746	5.30E-191
73	Y/N	+-	XZ-D_8_	gi|413955418	hypothetical protein ZEAMMB73_953540 [Zea mays]	21571.6/5.26	345	100	8	GRMZM2G314769		9:122457581–122458680	2.10E-22
75	3.5	+ -	XZ-D_8_	gi|413955418	hypothetical protein ZEAMMB73_953540 [Zea mays]	21571.6/5.26	165	100	4	GRMZM2G314769		9:122457581–122458680	2.10E-22
141	Y/N	A	LZ-D_8_	gi|413955418	hypothetical protein ZEAMMB73_953540 [Zea mays]	21571.6/5.26	185	100	7	GRMZM2G314769		9:122457581–122458680	2.10E-22
191	Y/N	A	XZ-D_10_	gi|413955418	hypothetical protein ZEAMMB73_953540 [Zea mays]	21571.6/5.26	173	100	4	GRMZM2G314769		9:122457581–122458680	2.10E-22
226	Y/N	+	LZ-D_10_	gi|413955418	hypothetical protein ZEAMMB73_953540 [Zea mays]	21571.6/5.26	92	99.99	4	GRMZM2G314769		9:122457581–122458680	2.10E-22
280	Y/N	A	LZ-D_12_	gi|413955418	hypothetical protein ZEAMMB73_953540 [Zea mays]	21571.6/5.26	95	99.994	5	GRMZM2G314769		9:122457581–122458680	2.10E-22
139	Y/N	-	LZ-D_8_	gi|226496343	uncharacterized protein LOC100273624 [Zea mays]	28169.5/6	187	100	7	GRMZM2G152258		2:231084849–231086151	5.00E-60
132	Y/N	A	LZ-D_8_	gi|194700784	unknown [Zea mays]	65656.1/6.31	103	100	16	GRMZM2G162688		5:189872033–189878985	3.10E-201
103	Y/N	-	XZ-D_8_	gi|194701170	unknown [Zea mays]	38872.7/7.52	441	100	6	GRMZM2G053206		2:232657306–232656057	2.00E-25
130	Y/N	-	LZ-D_8_	gi|194701170	unknown [Zea mays]	38872.7/7.52	228	100	5	GRMZM2G053206		2:232657306–232656057	2.00E-25
172	Y/N	-	XZ-D_10_	gi|194701170	unknown [Zea mays]	38872.7/7.52	503	100	7	GRMZM2G053206		2:232657306–232656057	2.00E-25
234	Y/N	-	XZ-D_12_	gi|223949117	unknown [Zea mays]	41017.8/5.28	136	100	11	GRMZM2G047564		9:88481641–88468222	3.50E-123
129	Y/N	A	LZ-D_8_	gi|223950161	unknown [Zea mays]	41693.3/6.32	306	100	11	GRMZM2G049811		9:49450407–49446403	2.10E-157
134	Y/N	A	LZ-D_8_	gi|223950161	unknown [Zea mays]	41693.3/6.32	273	100	14	GRMZM2G049811		9:49450407–49446403	2.10E-157
209	Y/N	+	LZ-D_10_	gi|223950161	unknown [Zea mays]	41693.3/6.32	441	100	18	GRMZM2G049811		9:49450407–49446403	2.10E-157
261	3.5	- -	XZ-D_12_	gi|223975961	unknown [Zea mays]	34782.7/5.59	657	100	14	GRMZM2G175562		6:88908479–88909745	1.50E-136
185	Y/N	A	XZ-D_10_	gi|219886829	unknown [Zea mays]	40504.2/4.99	225	100	14	GRMZM2G105019		3:217552207–217545545	1.20E-154
153	2.4	+	XZ-D_10_	gi|223975139	unknown [Zea mays]	57479.1/5.46	376	100	15	GRMZM2G161868		3:202435255–202437504	7.40E-256
85	Y/N	A	XZ-D_8_	gi|194699562	unknown [Zea mays]	50961.1/5.17	709	100	21	GRMZM2G372068		2:226478163–226476497	1.80E-101
264	Y/N	+ -	XZ-D_12_	gi|194699562	unknown [Zea mays]	50961.1/5.17	355	100	14	GRMZM2G372068		2:226478163–226476497	1.80E-101
203	Y/N	-	LZ-D_10_	gi|194692156	unknown [Zea mays]	39808.2/5.82	195	100	7	GRMZM2G055489		8:115838429–115832290	3.60E-112
293	Y/N	-	LZ-D_12_	gi|194692156	unknown [Zea mays]	39808.2/5.82	420	100	17	GRMZM2G055489		8:115838429–115832290	3.60E-112
192	Y/N	-	XZ-D_10_	gi|194702634	unknown [Zea mays]	24981.6/5.46	218	100	8	GRMZM2G096153		1:7863369–7862135	2.00E-49
215	Y/N	+ +	LZ-D_10_	gi|223947673	unknown [Zea mays]	34283.6/7.75	158	100	10	GRMZM2G014397		10:10731158–10736508	6.70E-120
68	Y/N	A	XZ-D_8_	gi|224029461	unknown [Zea mays]	28955.8/5.08	386	100	13	GRMZM2G148769		2:221504148–221501546	3.20E-88
136	Y/N	A	LZ-D_8_	gi|224029461	unknown [Zea mays]	28955.8/5.08	195	100	9	GRMZM2G148769		2:221504148–221501546	3.20E-88
181	Y/N	A	XZ-D_10_	gi|224029461	unknown [Zea mays]	28955.8/5.08	299	100	13	GRMZM2G148769		2:221504148–221501546	3.20E-88
256	Y/N	A	XZ-D_12_	gi|224029461	unknown [Zea mays]	28955.8/5.08	201	100	12	GRMZM2G148769		2:221504148–221501546	3.20E-88
276	Y/N	+	LZ-D_12_	gi|224029461	unknown [Zea mays]	28955.8/5.08	238	100	9	GRMZM2G148769		2:221504148–221501546	3.20E-88
167	2.6	+	XZ-D_10_	gi|194697638	unknown [Zea mays]	64089/4.8	270	100	16	GRMZM2G048324		1:70432507–70436832	8.00E-156
97	Y/N	+	XZ-D_8_	gi|218319054	unnamed protein product [Zea mays]	27952.1/5.47	509	100	11	GRMZM2G038075		5:3146159–3147709	1.50E-74
125	Y/N	+	LZ-D_8_	gi|218319054	unnamed protein product [Zea mays]	27952.1/5.47	346	100	10	GRMZM2G038075		5:3146159–3147709	1.50E-74
205	5.8	A	LZ-D_10_	gi|218319054	unnamed protein product [Zea mays]	27952.1/5.47	161	100	10	GRMZM2G038075		5:3146159–3147709	1.50E-74
250	Y/N	-	XZ-D_12_	gi|257737930	unnamed protein product [Zea mays]	37624.5/4.98	178	100	8			1:77358025–77358598	1.60E-93
206	Y/N	-	LZ-D_10_	gi|295415566	unnamed protein product [Zea mays]	34182/5.91	359	100	14	GRMZM2G005887		1:177054888–177059486	2.80E-128
230	Y/N	+	LZ-D_10_	gi|295421207	unnamed protein product [Zea mays]	34240/5.68	420	100	17	GRMZM2G005887		1:177054888–177059486	1.90E-129
92	Y/N	+	XZ-D_8_	gi|296515149	unnamed protein product [Zea mays]	29489.9/5.76	779	100	16	GRMZM2G038075		5:3146159–3147709	3.10E-74
271	Y/N	+ -	XZ-D_12_	gi|296515149	unnamed protein product [Zea mays]	29489.9/5.76	262	100	13	GRMZM2G038075		5:3146159–3147709	3.10E-74
175	Y/N	A	XZ-D_10_	gi|298547953	unnamed protein product [Zea mays]	53307.3/5.92	516	100	19	GRMZM2G145715		3:153967866–153964586	2.90E-238
142	4.3	+	XZ-D_10_	gi|257696214	unnamed protein product [Zea mays]	39454.2/7.12	402	100	12	GRMZM2G137839		1:43682852–43685890	1.50E-113
143	4.9	+	XZ-D_10_	gi|257696214	unnamed protein product [Zea mays]	39454.2/7.12	479	100	15	GRMZM2G137839		1:43682852–43685890	1.50E-113
170	Y/N	+	XZ-D_10_	gi|257640662	unnamed protein product [Zea mays]	39196.1/6.09	684	100	19	GRMZM2G328094		8:6457224–6458504	1.90E-113
208	Y/N	+	LZ-D_10_	gi|257640662	unnamed protein product [Zea mays]	39196.1/6.09	388	100	17	GRMZM2G328094		8:6457224–6458504	1.90E-113
249	Y/N	A	XZ-D_12_	gi|257676371	unnamed protein product [Zea mays]	23715.2/4.65	175	100	3	GRMZM2G175076		3:231979651–231978427	2.80E-41
82	Y/N	A	XZ-D_8_	gi|257333572	unnamed protein product [Zea mays]	64533.6/6.13	498	100	22	GRMZM2G016890		10:34240678–34245632	1.10E-128
83	9.5	A	XZ-D_8_	gi|257333572	unnamed protein product [Zea mays]	64533.6/6.13	658	100	23	GRMZM2G016890		10:34240678–34245632	1.10E-128
84	Y/N	+	XZ-D_8_	gi|257333572	unnamed protein product [Zea mays]	64533.6/6.13	557	100	23	GRMZM2G016890		10:34240678–34245632	1.10E-128
154	Y/N	A	XZ-D_10_	gi|257333572	unnamed protein product [Zea mays]	64533.6/6.13	260	100	18	GRMZM2G016890		10:34240678–34245632	1.10E-128
195	Y/N	-	LZ-D_10_	gi|257333572	unnamed protein product [Zea mays]	64533.6/6.13	468	100	26	GRMZM2G016890		10:34240678–34245632	1.10E-128
294	Y/N	-	LZ-D_12_	gi|257333572	unnamed protein product [Zea mays]	64533.6/6.13	372	100	19	GRMZM2G016890		10:34240678–34245632	1.10E-128
196	Y/N	-	LZ-D10	gi|257714156	unnamed protein product [Zea mays]	64551.6/6.23	360	100	22	GRMZM2G016890		10:34240678–34245632	4.60E-128
295	Y/N	-	LZ-D_12_	gi|257714156	unnamed protein product [Zea mays]	64551.6/6.23	334	100	17	GRMZM2G016890		10:34240678–34245632	4.60E-128
77	Y/N	A	XZ-D_8_	gi|296511817	unnamed protein product [Zea mays]	46789.3/5.45	722	100	19	GRMZM2G084881		2:166253571–166248891	6.90E-171
78	Y/N	A	XZ-D_8_	gi|219972638	unnamed protein product [Zea mays]	46039.3/5.21	562	100	13	GRMZM2G098239		2:10633629–10631020	2.40E-60
253	3.9	- -	XZ-D_12_	gi|257725861	unnamed protein product [Zea mays]	60702.2/5	386	100	22			6:142273504–142277935	7.60E-215
93	Y/N	A	XZ-D_8_	gi|219746454	unnamed protein product [Zea mays]	25750.4/5.52	282	100	10	GRMZM2G434541		3:206915357–206915753	2.70E-52

Notes

^a^ Spot No. corresponds to labels in 2-DE map.

^b^ Maximum fold changes among three groups (female line, male line, and hybrid combination) were calculated by ANOVA.Y/N means that difference between them was present or absent.

^c^ Heterotic patterns were examined following Hoecker et al. (2008). “A” indicates protein spots that had no significant difference in average spot intensities with midparent value at 0.05 level. Average spot intensities of proteins that deviated significantly from the mid-parent value of the parental lines at the 0.05 cut-off level were defined as non-additive proteins. Based on this premise, “+” and “-” represented protein spot intensities identified in F_1_ hybrids that were similar to the high parent and low parent values, respectively. “+ +” and “- -” represented the protein spot intensities identified in F_1_ hybrids that were significantly different from the high parent and the low parent values, respectively. “+ -” represented the protein spot intensities identified in F_1_ hybrids that fell in between the mid-parent and the high parent or the mid-parent and the low parent values.

^d^ Hybrids Xun928×Zong3 and Lx9801×Zong3 are abbreviated as XZ and LZ, respectively. D_8_, D_10_, and D_12_ represent sampling stages.

^e^ GenBank accession number of protein spot.

^f^ Protein name in NCBI database.

^g^ Proteins scores were derived from ions scores as a non-probabilistic basis for ranking protein hits.

^h^ Confidence interval of the identified protein.

^i^ Gene name retrieved from maize sequence (http://ensembl.gramene.org/Zea_mays/Info/Index) by cDNA blast.

^j^ Physical position determined by blast function in MaizeGDB.

### Differentially accumulated proteins identified as important for silk viability and its heterosis

Three proteins differentially accumulated during silk development were identified, including gi|413944345 (protein spot 63 in Zong3), gi|414869037 (protein spot 8 in Xun928), and gi|195635735 (protein spot 16 in Xun928). These three proteins also differentially regulated the heterosis of silk viability in the two hybrids (Tables 2 and 3). gi|413944345, which differentially regulated silk development in the common paternal line Zong3, showed differential accumulation in the two hybrids Xun928×Zong3 and Lx9801×Zong3 at almost all sampling stages. gi|414869037 and gi|195635735, which were specific for silk development in the inbred line Xun928, contributed to the heterosis of silk viability only in the hybrid Xun928×Zong3 (protein spots 155 and 239 at D_10_ and D_12_; protein spot 96 at D_8_), and not in Lx9801×Zong3. The functional category analysis showed that these three proteins were involved in anthocyanin biosynthesis, methionine metabolism, and suberin biosynthesis.

Additionally, the proteins gi|195643366 (APx2, cytosolic ascorbate peroxidase; protein spots 74, 190, 268), gi|413942605 (6-phosphogluconate dehydrogenase isoenzyme B, protein spots 104, 176, 247), and gi|413920184 (O-methyltransferase ZRP4, protein spots 89,150, 272) regulated the heterosis of silk viability at all sampling stages only in the hybrid Xun928×Zong3. The proteins gi|414878829 (glutathione S-transferase 4, protein spots 127, 216, 298) and gi|414881303 (anthocyaninless1, protein spots 138, 224, 285), affected the heterosis of silk viability at all sampling stages only in the hybrid Lx9801×Zong3 (Table 3). Three out of these five proteins were involved in secondary metabolism in the phenylpropanoid pathway. The proteins gi|195629642 (lichenase-2 precursor, protein spots 267, 290) and gi|413920639 (chitinase 1, protein spots 255, 275) contributed to silk viability heterosis only at the late silk developmental stage (D_12_) of the two hybrids.

### Functional category and KEGG pathway enrichment classifications of differentially accumulated proteins

**Fig 1:**
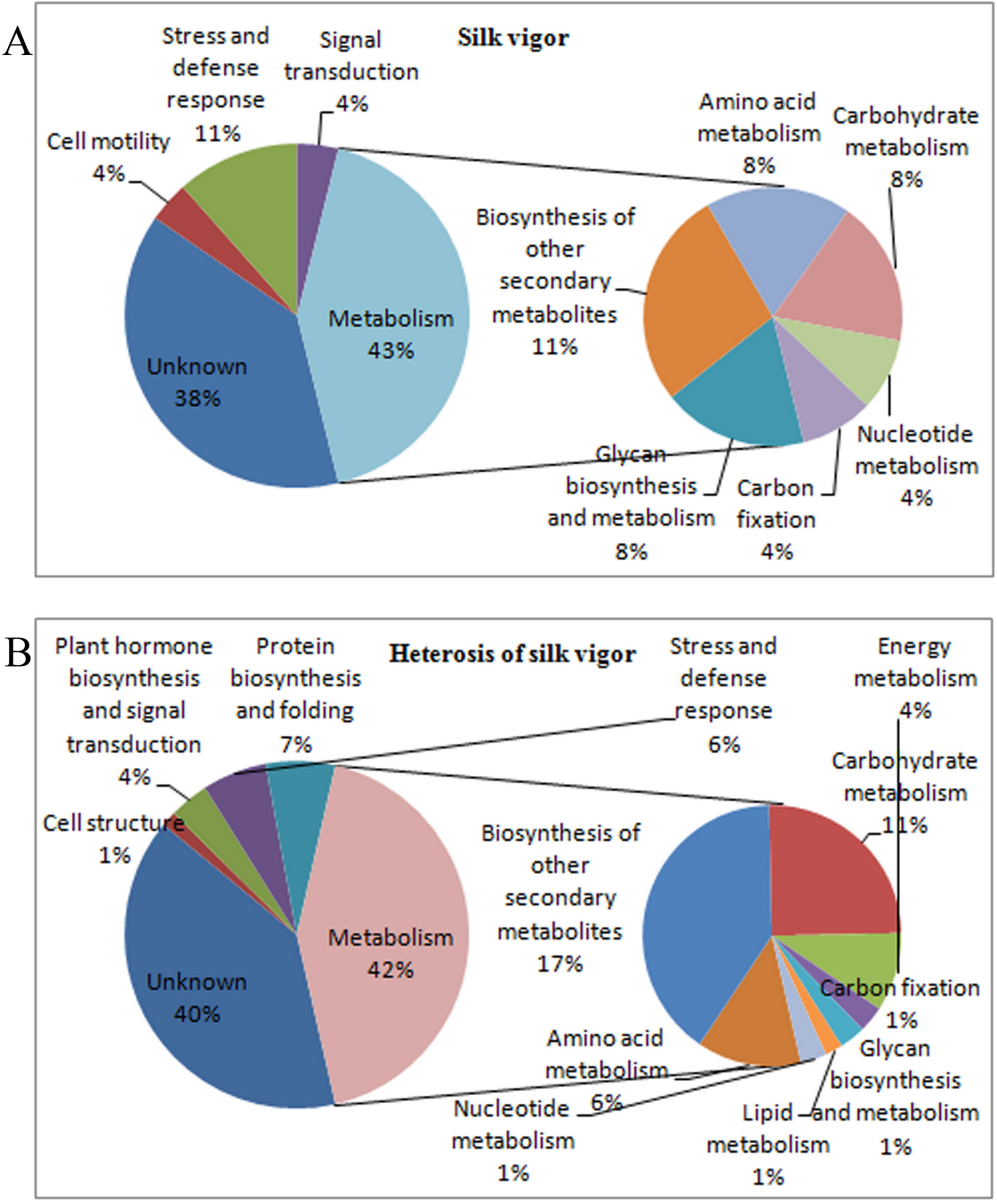
Functional categories of differentially accumulated proteins in the inbred lines and its corresponding hybrid combinations.

The differentially accumulated proteins associated with silk viability and its heterosis was in similar functional categories. Unknown proteins comprised a large proportion of the differentially accumulated proteins, accounting for 38% and 40% of the proteins related to silk viability and its heterosis, respectively. The proteins involved in metabolism group accounted for the largest proportion of the differentially accumulated proteins, accounting for 43% of proteins related to silk viability and 42% of proteins related to the heterosis (Fig 1). Proteins involved in protein biosynthesis and folding, including transcription, translation, folding, sorting and degradation, were the second most abundant group and were specific to the heterosis of silk viability. Other important categories, based on protein abundance, were stress and defense response, plant hormone biosynthesis and signal transduction, and cellular processes. In the largest category, metabolism, there were six and eight subcategories of proteins involved in silk viability and its heterosis, respectively. Among them, methionine metabolism and flavonoid metabolism were important for both silk viability and its heterosis (Tables 2 and 3, Fig 1), and lipid metabolism and energy metabolism were specific to the heterosis of silk viability.

**Fig 2:**
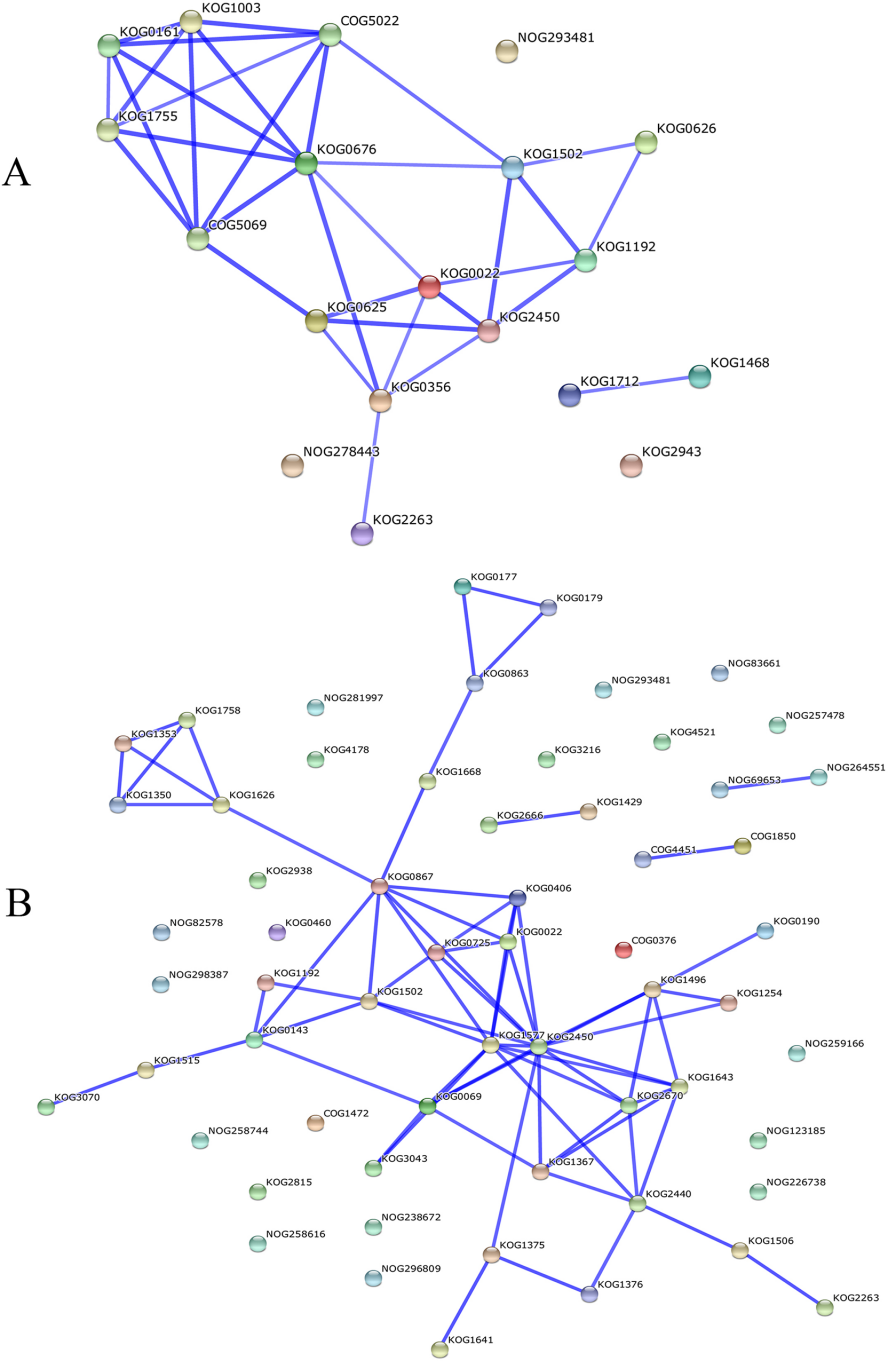
Protein–protein interaction networks obtained *in silico* using String database, with COG functions.

The protein–protein interaction networks involved in silk viability and its heterosis were analyzed by searching the String database (Fig 2). Three proteins were implicated in silk viability and its heterosis: gi|413944345 (KOG1192), gi|414869037 (KOG2263) and gi|195635735 (NOG293481). These proteins had only one or two interacting proteins and were distributed at a remote node in the network. Some reductases or dehydrogenases (S4 Table) were located at the interaction nodes and played an important role in the protein–protein interaction networks both for silk viability and its heterosis; for example, KOG1502-KOG2450-KOG0022 in the interaction network for silk viability and KOG1502-KOG1577-KOG2450-KOG0022-KOG0725 in the interaction network for the heterosis of silk viability. The two important branches for the heterosis of silk viability were ATP energy production (KOG1758-KOG1353-KOG1350-KOG1626-) and protein metabolism (KOG0177-KOG0179-KOG0863-KOG1688-) (Fig 2B). Additionally, two glutathione S-transferases (gi|195619648 KOG0406, gi|162460516 KOG0867) might play a crucial role in providing energy and proteins for the entire protein–protein interaction network for the heterosis of silk viability. Meanwhile, Kinases (KOG1367, KOG2440), enolase (KOG2670), and isomerase (KOG1643) increased the complexity of the protein–protein interaction network for the heterosis of silk viability compared with the network for silk viability, which was complicated by cell cytoskeleton proteins (Fig 2A).

## Discussion

### Comparison of protein categories related to phenotypes of the inbred lines and their corresponding hybrids

The results in this study revealed that several functional categories of proteins corresponded to the seed setting rate phenotype of the inbred lines and its corresponding hybrids. For the inbred lines, proteins involved in flavonoid metabolism, methionine metabolism and cytokinin signaling, made the highest contributions to contributed the highest silk viability in the inbred line Zong3 (Table 2). Compared to the inbred line Lx9801, the stronger silk viability of the inbred line Xun928 was attributed to proteins involved in methionine metabolism, and these proteins also contributed to the heterosis of silk viability in the hybrids, but the proportions of their contributions differed. Proteins involved in flavonoid metabolism were important for heterosis of silk viability at all sampling stages in the two hybrids (Table 3). However, proteins involved in methionine metabolism contributed differently to the heterosis of silk viability at different developmental stages of silks in the two hybrids: at D_10_ and D_12_ for Xun928×Zong3, and at D_8_ and D_10_ for Lx9801×Zong3. These results implied that proteins contributing to silk viability were not always as important for the heterosis of silk viability in the hybrids. Compared with the hybrid Lx9801×Zong3 at the three sampling stages, the hybrid Xun928×Zong3 accumulated more proteins involved in protein biosynthesis and folding, stress and defense responses, signal transduction and cell detoxification in response to genetic and environmental changes. Thus, the hybrid Xun928×Zong3 showed stronger heterosis than the hybrid Lx9801×Zong3.

### Potential regulation networks revealed by differentially accumulated proteins related to silk viability and its heterosis

#### Methionine metabolism and salvage cycle

Nutrient supply is a basic requirement for successful fertilization. The nutrients in pollen, however, can support only about 2 cm of tube growth in the maize silk [21]. Thus, the maize silk must provide enough nutrients to support pollen tube growth over a longer distance. Consistent with this, many differentially accumulated proteins were related to cysteine and methionine metabolism (Tables 2 and 3).

Proteins involved in methionine supply were important for silk viability. Methionine functions not only as a building block for protein synthesis, but also as a signaling molecule in communicating intracellular metabolic events to receptors on the cell surface. Therefore, methionine could supply appropriate signals to support pollen tube growth and guidance in the maize silk. In plants, methionine synthase (MeSe EC 2.1.1.12; protein spots 8, 121, 155, 239) catalyzes the terminal step of the methionine synthesis pathway by transferring a methyl group to homocysteine (Hcy), producing methionine. However, this *de novo* synthesis is energetically expensive and highly tissue-specific. To save energy consumption, about 80% of the methionine is recycled [22]. S-Adenosylmethionine (AdoMet)-dependent transferase (protein spots 148, 194, 238) plays a critical role in methionine recycling by transferring the methyl group from AdoMet to S-adenosylhomocysteine (AdoHcy). This is not the only methionine recycling pathway in plants (S3 Fig). Methylthioribose-1-phosphate isomerase (protein spot 64), which catalyzes the phosphorylated methylthioribose (MTR) to methylthioribulose-1-P, is the first and ubiquitous enzyme for methionine recycling. The accumulation of adenine (Ade), a by-product of MTR formation, inhibits methionine recycling. On the other hand, Ade is also a substrate for phosphoribosyl Transferase 1 (APT1) (protein spot 46), which regulates cytokinin levels by converting active cytokinin forms to inactive ones. Loss of APT1 activity leads to excess accumulation of cytokinins, inducing a myriad of cytokinin-regulated responses, such as delayed leaf senescence, anthocyanin accumulation, and downstream gene expression [23].

AdoMet, as the major product of methionine metabolism, is an important cofactor that modulates various biological activities [24,25]. As the major methyl-group donor, AdoMet can regulate transmethylation reactions at the levels of DNA metabolism, RNA metabolism, and protein post-translational modifications [26]. AdoMet is also involved in metabolic and developmental regulation, since it is a substrate for thesynthesis of nicotianamine, ethylene (1-aminocyclopropane-1-carboxylate synthase), and polyamines [22]. AdoMet metabolism is complicated by its interaction with plant growth hormones such as cytokinins and auxins [27]. Thus, AdoMet is involved in regulating plant developmental by fine-tuning gene transcription, cell proliferation, and the production of secondary metabolites [28,29].

In this study, positive regulators of nutrients production were identified in the inbred lines Zong3 (protein spots 46, 64) and Xun928 (protein spot 8), but not in the inbred line Lx9801. In the hybrid combinations, relatively more positive regulators (protein spots 148, 155, 238, 239) were identified at the late developmental stages (D_10_ and D_12_) in Xun928×Zong3. However, only two positive regulators (protein spots 121, 194) were identified in hybrid Lx9801×Zong3 and differentially accumulated at D_8_ and D_10_. These results were consistent with the stronger silk viability of the inbred lines Zong3 and Xun928 than that of Lx9801, as well as the high seed setting rates (84.6% for D_10_ and 80.2% for D_12_) and mid-parent heterotic degrees (34.4% for D_10_ and 41.4% for D_12_; data not shown) during the late sampling stages in the hybrid Xun928×Zong3. For comparison, Lx9801×Zong3 showed seed setting rates of 70.4% and 66.9% at D_10_ and D_12_; and mid-parent heterotic degrees of 21.1% and 31.3% at D_10_ and D_12_ (Tables 1–3).

#### Photosystem and energy metabolism

Photosynthesis provides fuel for plant growth by converting light energy into chemical energy. Ribulose-1,5-bisphosphate carboxylase/oxygenase (RuBisCO), catalyzes the first major step of the Calvin cycle (carbon fixation) to produce energy-rich carbohydrates. This reaction uses ATP as an energy source and NADPH as reducing power, and is often the rate-limiting step in photosynthesis [30]. In all eukaryotes, Rubisco is an oligomer consisting of eight large subunits bound to eight small subunits. The large subunits (protein spots 67, 106, 178) contain the enzymatically active substrate binding sites and are synthesized in the chloroplast. The small subunits are synthesized in precursor form by cytoplasmic ribosomes. Assisted by the RuBisCO large subunit-binding protein (protein spot 40), mature small subunits assemble with large subunits to form the oligomeric holoenzyme in the stroma. Several studies have shown that increased expression levels of RuBisCO subunits could increase photosynthetic efficiency by increasing catalytic activity and/or by decreasing the oxygenation rate [31].

ATP synthase (EC 3.6.3.14), a key enzyme in energy metabolism, is widely involved in oxidative and photosynthetic phosphorylation and plays an important role in many processes in plants. It consists of two rotary motors: the membrane-integrated CF_o_ and the hydrophilic CF_1_. CF_o_ mainly participates in proton transport through thylakoids, whereas CF_1_ contains the nucleotide binding, catalytic, and regulatory sites of the ATP complex [32]. CF_1_ contains five subunits: α (protein spot 199), β, γ, δ (protein spot 182), and ε [32,33]. The gene encoding the CF_1_ α subunit, *atpA*, was shown to be related to cold resistance, and the transcript level of *atpA* was positively correlated with ATP synthase activity [34]. Mutation of the *atpA* gene in a cytoplasmic male sterile line caused an energy supply shortage during flower development, resulting in abnormal microspore development compared with its maintainer [35].

In this study, proteins involved in energy metabolism differentially accumulated in the inbred line Zong3 (protein spot 40) and the hybrid Xun928×Zong3 at D_8_ and D_10_ (protein spots 67, 106, 178, 182). A sufficient energy supply may be important to support stronger silk viability of Zong3, compared with those of Xun928 and Lx9801, and the stronger heterosis of silk viability in Xun928×Zong3 than in Lx9801×Zong3.

#### Protein metabolism and cell senescence

Proteins have a vast array of functions within living organisms, including catalyzing metabolic reactions, replicating DNA, responding to stimuli, and transporting molecules from one location to another. Many elaborate regulation mechanisms are involved in converting DNA sequences into functional proteins. Translation, the assembly of proteins by ribosomes, is an essential part of the protein biosynthetic pathway and requires initiation and elongation complexes [21]. Eukaryotic translation initiation factor 5A (eIF-5A) (protein spots 72, 146) not only regulates protein synthesis but also acts as an important determinant of cell proliferation and senescence. In dividing and dying cells, different isoforms of eIF-5A execute its biological switching function in response to physiological and environmental cues [36–38]. The elongation factor-1 (EF1) complex (protein spots 112, 161, 251) is responsible for the enzymatic delivery of aminoacyl tRNAs to the ribosome. EF1A is responsible for the selection and binding of the cognate aminoacyl-tRNA to the acceptor site of the ribosome. EF1 delta (protein spots 112, 251), functions as a guanine nucleotide exchange factor in regenerating active EF1A-GTP from inactive EF1A-GDP. During and after protein synthesis, polypeptide chains often fold into their native secondary and tertiary structures, whether they are used in the cell or secreted. To achieve their final correct states, cellular and secreted proteins require the help of several other folding proteins or chaperones. Protein disulfide isomerase (protein spot 252) catalyzes protein-folding, allowing proteins to reach their final correctly folded state without enzymatic disulfide shuffling [39]. Unneeded or damaged proteins are transferred to proteasomes, an active complex composed of α subunits and β subunits (protein spots 229, 288), to be degraded into amino acids that are used to synthesize new proteins. At all sampling stages, many differentially accumulated proteins involved in protein biosynthesis and correct folding were identified in the hybrid Xun928×Zong3. However, more proteins involved in proteasomes differentially accumulated during the late sampling stages (D_10_ and D_12_) in the hybrid Lx9801×Zong3. These results implied that the hybrid Lx9801×Zong3 might consume more resources during normal metabolism, which weakened its silk viability, especially at the late silk developmental stages.

During normal plant development, the insoluble polyesters suberin and cutin form extracellular lipophilic barriers to prevent membrane leakiness [40]. However, membranes become leaky when the cell begins to senescence. This process is usually accompanied by the accumulation of proteins involved in lipid metabolism. In this study, O-methyltransferase (protein spots 89, 150, 163, 171, 236, 243, 272), the first rate-limiting enzyme in suberin synthesis, and GDSL-motif lipase/hydrolase (protein spot 96), an enzyme involved in the hydrolysis and transfer of activated monomers in cutin synthesis [41], differentially accumulated in the hybrid Xun928×Zong3. Proteins involved in lipid metabolism only differentially accumulated in the hybrid Lx9801×Zong3. These results implied that membrane leakiness might occur earlier in Lx9801×Zong3 than in Xun928×Zong3 at the late silk developmental stages. Thus, silk viability was lost earlier in Lx9801×Zong3 than in Xun928×Zong3. This pattern of protein accumulation might also explain the faster decrease in the seed setting rate and the weaker heterotic degree in the hybrid Lx9801×Zong3 at the late silk developmental stages.

#### Phenylpropanoid metabolism and plant hormones regulation

The plant hormone auxin regulates cell elongation, division, differentiation, and morphogenesis. Many proteins involved in elaborate temporal and spatial regulation of auxin metabolism, transport, and signaling have been identified. Auxin-binding protein 1 (ABP1: protein spot 193) mediates cell elongation and, directly or indirectly, cell division. In previous studies, ectopic and inducible expression of ABP1 conferred auxin-dependent cell expansion in tobacco cells that normally lack auxin responsiveness [42] and antisense suppression of ABP1 eliminated auxin-induced cell elongation and reduces cell division. A homozygous null mutation of ABP1 was embryo-lethal in *Arabidopsis* [43]. Auxin-induced swelling of proteoplasts and intact guard cells can also be attributed to ABP1 [44,45]. Pyrophosphate-energized vacuolar membrane proton pump 1 (protein spot 69) facilitates auxin transport and regulates auxin-mediated developmental processes by modulating apoplastic pH [46]. Flavonoids in the phenylpropanoid pathway are another regulator of active auxin and have species-specific roles in nodulation, fertility, defense, and ultraviolet protection. Flavonols have been shown to negatively regulate the polar auxin transport (PAT) by competing for free auxin with auxin efflux carriers such as PIN and ABCB (PGP proteins) in vivo [47]. Dihydroflavonol 4-reductase (DFR4, EC1.1.1.219: protein spot 53) and UDP-glucoside: flavonoid glucosyltransferase (EC 2.4.1.115: protein spots 63, 76, 79, 99, 117, 119, 122, 123, 138, 152, 159, 188, 197, 200, 201, 202, 212, 224, 231, 241, 285) are the first and last enzymes in the anthocyanin biosynthetic pathway, respectively. Their differential accumulation may be related to competition for the dihydroflavonol substrate with the flavonol branch, and thus, could indirectly affect PAT. The relatively higher contents of anthocyanin biosynthetic enzymes corresponded to higher levels of glutathione S-transferase-like proteins (protein spots 127, 216, 217, 298), which transport anthocyanins from the ER to the vacuole [48] in the hybrid Lx9801×Zong3 at the three sampling stages.

Cytokinin is another important hormone that regulates cell proliferation and differentiation. The two active forms of cytokinins are the isopentenyl adenine (iP)-type and the zeatin-type [49]. The metabolic regulation of cytokinin includes biosynthesis, interconversion, inactivation and degradation [50]. β-glucosidase (EC 3.2.1.21, protein spot 61) encoded by *Zm-p60*.*1* catalyzes the release of active cytokinins from their inactive storage and transport forms (cytokinin-O-glucosides) [51]. Over-expression of *Zm-p60*.*1* disrupted zeatin homeostasis in intact transgenic plants, rendering them hypersensitive to exogenous zeatin [51]. Inactive cytokinin can also be generated by cytokinin-O-glucosyltransferase (protein spot 107) [52,53]. Studies of maize transformants harboring zeatin O-glucosyltransferase have shown that zeatin O-glucosylation affects root formation, leaf development, chlorophyll content, senescence, and male flower differentiation through developmental modifications [53].

Proteins involved in the regulation of hormone levels (ABP1, pyrophosphate-energized vacuolar membrane proton pump 1; anthocyanin biosynthesis pathway; and cytokinin-O-glucosyltransferase) were identified as being important for both silk viability and its heterosis. These proteins differentially accumulated in the hybrid Xun928×Zong3, whereas only those involved in anthocyanin biosynthesis differentially accumulated in the hybrid Lx9801×Zong3. The flexibility of the systems regulating hormone levels may explain the increase in silk viability in the hybrid Xun928×Zong3, resulting in the high seed setting rate and strong heterosis.

In summary, proteins gi|413944345, gi|414869037, and gi|195635735 were attractive and might be related with silk viability as well as its heterosis. Significant correlation (*r* = 0.827^*^ for gi|413944345; *r* = -0.365^*^ for gi|414869037; *r* = 0.556^*^ for gi|195635735) was detected between these protein spots accumulation level and seed setting rate. Thus, we could propose the following hypotheses regarding proteins related to silk viability and its heterosis: methionine salvage, protein synthesis, and ATP supply function as positive regulators of silk viability, and therefore, contribute to strong silk viability and its heterosis in Zong3 and Xun928×Zong3, respectively. Active fatty acid metabolism, a signal for cell wall degradation, and anthocyanins, which negatively regulate local hormone accumulation, were related to weaker silk viability in Lx9801 and Lx9801×Zong3. The metabolism of cutin and suberin, which were derived from phenylpropanoid precursors, might confer stronger silk viability (Zong3 and Xun928) and stronger heterosis of silk viability in hybrids by slowing the silk aging process, especially during the late stages of silk development.

## Conclusions

In this study, the heterosis of silk viability could be mainly attributed to additive accumulation of differentially regulated proteins, although proteins that accumulated in a non-additive manner made a similar contribution. Simple additive and dominant effects at a single locus, as well as complex epistatic interactions of metabolic pathway genes at two or more loci, resulted in partially dominant silk viability heterosis in the hybrids. For silk viability, most important differentially accumulated proteins were those involved in methionine metabolism for nutrient supply, phenylpropanoid metabolism for hormone homeostasis, protein biosynthesis and metabolism for genetic information processing, and carbon fixation for energy generation.

## Materials and Methods

### Plant materials

Three typical inbred lines, Zong3, Xun928, and Lx9801, with different silk viability were used in this study. Among more than one hundred inbred lines, the silk viability of the inbred line Zong3 was extremely high. The inbred lines Xun928 and Lx9801 had relatively weak silk viability. To assay the heterosis of silk viability in different genetic backgrounds, two hybrids, Xun928×Zong3 and Lx9801×Zong3 were created in this study. The two hybrids and the three inbred lines were planted on the farm of Henan Agricultural University (Zhengzhou, China; E 113°42′, N 34°48′) in summer of 2013, when the daily average temperature was 14.3°C. The annual average rainfall is 640.9 mm in this region. Each plot consisted of ten 5-m-long rows, with 20 cm of in-row spacing and 67 cm of inter-row spacing. Only the middle rows were sampled to avoid edge effects. Before the silks emerged from the husk, ear shoots were totally covered with bags to avoid pollen contamination. To evaluate the silking time accurately, the silking time of each ear of the materials was recorded in the field. Day 1 (D_1_) was marked as the day that the silks emerged above the ligule of the outer leaf of the husk. Silks were removed from the mid-base region of each ear at D_4_, D_6_, D_8_, D_10_, and D_12_ and immediately frozen in liquid nitrogen in the field. Each sample was collected with three biological replications and 10 ears were mixed for each replication. At the same time, the ear for each sample was saturation-pollinated by hand on the sampling day to measure the seed setting rate. Pollination was completed between 9 and 10 a.m. (below 37°C) to ensure consistent pollination efficiency. The ears were harvested at physiological maturity, and only the seeds at the mid-base (5–15 rounds from the base) were used to calculate the seed setting rate of the cob according to silk development characteristics [54]. The seed setting rate was calculated by dividing the total number of spikelets by the number of fully grown seeds.

### Protein extraction and MS identification

Each genotype was assayed with three biological replications corresponding to each sampling stage. Frozen silks (1 cm, approx. 1.0 g) of each biological replication (mixture of silks from ten different plants) were fully ground in liquid nitrogen and then extracted in 10 mL pre-cooled trichloroacetate (TCA) buffer (10% w/v TCA in acetone with 0.07% β-mercaptoethanol) with vortexing for 2 h at 20°C. After centrifugation at 15,000 × g for 30 min, the supernatant was discarded and the precipitate was rinsed with 10 mL chilled buffer (80% acetone with 0.07% β-mercaptoethanol) four times by centrifuging for 10 min at 15,000 × g. The final cleaned precipitate was freeze-dried under a vacuum. The dried protein pellet per 1 mg was resuspended in 20 μL buffer (8 M urea, 2 M thiourea, 4% (w/v) CHAPS and 40 mM dithiothreitol (all from Solarbio)). The protein was quantified using a Bio-Rad protein assay with bovine serum albumin as a standard and used for two-dimensional gel electrophoresis (2-DE). Three technical replications were assayed for each biological replication. For each technical replication, equal amounts of total protein extract (800 μg) were used for isoelectric focusing (IEF). Immobilized dry strips (24 cm, Imobiline drystrips, Bio Rad, Hercules, CA, USA) with a linear gradient of pH 4–7 were rehydrated for 16 h at 50 V. The IEF conditions for separating proteins were as followes: slow 250 V for 30 min, rapid 250 V for 2 h, rapid 500 V for 2 h, rapid 1,000 V for 2 h, linear 9,000 V for 5 h, rapid 10,000 V for 10 h, and a constant 500 V for the final 12 h at 20°C. Strips were immediately equilibrated in 10 mL of two types of SDS equilibration buffer for 15 min each. Buffer 1 contained 0.375 M Tris-HCl pH 8.8, 6 M urea, 20% glycerol, 4% SDS, and 2% DTT and buffer 2 contained 0.375 M Tris-HCl pH 8.8, 6 M urea, 20% glycerol, 4% SDS, and 2.5% iodoacetamide. IPG gel strips with the proteins were embedded into the top of a polyacrylamide gel (12%) after equilibration and separated at a constant voltage of 50 V for 30 min. Then, a constant voltage of 200 V was maintained until the electrophoresis was finished.

Digital images of the gels stained with Coomassie brilliant blue G250 were obtained with a scanner (UMAX Power Look 2100 XL). Spot detection and matching was performed with the default parameters using the “spot detection wizard” function in PDQuest 8.0 software. The “find spot centers” function was used with default auto-noise smoothing and background subtraction. A Gaussian model was selected to generate a master gel for each image file. All the gels were matched to the reference master gels selected and normalized in automated mode followed by manual group correction. The normalization parameters were “total quantity in valid spots”, “total density in gel image”, “mean of log ratios”, and “local regression model”. After normalization, ANOVA was used to calculate the significance of differences in the relative abundance of protein in individual spot features among the developmental stages of a certain inbred line, as well as among hybrids and their corresponding inbred lines at each developmental stage. For protein spots further assayed by MS, the maximum intensity variation criterion was set to ≥ 1.5-fold and ≥ 2-fold (*P* < 0.05) among different sampling stages for each inbred line, and between hybrid and parental inbred lines at each sampling stage, respectively.

The selected proteins were excised manually from gels, subjected to in-gel digestion with trypsin, and then destained using 25 mM ammonium bicarbonate in 50% (v/v) acetonitrile for 15 min at room temperature. The discolored spots were vacuum-dried and incubated with modified porcine trypsin at 37°C overnight. After centrifugation, the supernatant was collected and vacuum-dried, and then the precipitate was re-dissolved in 60% acrylonitrile/0.1% trifluoroacetic acid (TFA) (100 μL) for 15 min to obtain the peptides. Then, a 0.3 mL peptide sample and 0.3 mL matrix consisting of 10 mg/mL α-cyano-4-hydroxycinnamic acid in 50% acetonitrile and 0.1% TFA was analyzed on a matrix-assisted laser desorption ionization-time of flight (MALDI-TOF) mass spectrometry (MS). The parameters of the MS were set with 4000 Series Explorer software (Applied Biosystems). The lists of theoretical peptide MS from each peptide-map-fingerprinting (PMF) combined with MS/MS were used to search the NCBI (National Center for Biotechnology Information) database without repetition for homologous sequences using MASCOT 2.2 software (www.matrixscience.com). The search criteria were as follows: 1) peptide mass tolerance of 100 ppm; 2) maximum of a single missed tryptic cleavage; 3) fragment mass tolerance of 0.4 Da; and 4) carbamidomethylation by cysteine residues as fixed modifications and oxidation by methionine residues as dynamic modifications. Only proteins with a MASCOT score > 60 with 95% confidence and at least two matched peptides were accepted. Gene Ontology (GO) annotations and the theoretical Mr/pI for the identified proteins were retrieved from http://www.geneontology.org/ and http://www.expasy.ch/tools/pi_tools.html, respectively. Kyoto Encyclopedia of Genes and Genomes (KEGG) pathway enrichment analysis was carried out using the blast function in BLAST2GO. The protein–protein interaction network was analyzed by the publicly available program STRING (http://string-db.org/). Clusters of Orthologous Groups (COG) of proteins functions were used to construct the networks. Only an interaction networks with a high confidence (0.700 for silk viability or 0.900 for heterosis of silk viability) and no more than five interactors were retained. The eukaryotic orthologous groups (KOGs) were considered prime selections for a single protein spot.

### Data analysis

Protein spots that had no significant difference in average spot intensity from the mid-parent value at the 0.05 level were considered additively accumulated (A). The accumulation pattern of each non-additive protein was classified as described by Hoecker et al. [55]. Average spot intensities of proteins that deviated significantly from the mid-parent value of the parental lines at *P* < 0.05 level were defined as non-additive proteins. “+” and “−” were used to indicate that the protein spot intensity identified in the F_1_ hybrid was similar to the high parent and low parent values, respectively. “+ +” and “− −” indicated that the protein spot intensity identified in the F_1_ hybrid was significantly different from the high parent and low parent values, respectively. “+ −” indicated the protein spot intensity identified in the F_1_ hybrid fell in between the mid-parent and high parent or mid-parent and low parent values. ANOVA, LSD, and correlation analysis were performed with the corresponding function in Excel 2007.

## Supporting Information

S1 Fig Representative 2-DE map of proteins related to silk viability at different developmental stages. (DOCX)

S2 Fig Representative 2-DE map of proteins related to the heterosis of silk viability in the hybrid combinations of Xun928×Zong3 and Lx9801×Zong3 at different developmental stages. (DOCX)

S3 Fig Recycling of methionine cited from Ravanel et al. [28]. Enzymes: 1, cystathionine g-synthase; 2, cystathionine b-lyase; 3, methionine synthase; 4, AdoMet synthetase; 5, AdoMet-dependent methylase; 6, AdoHcy hydrolase; 7, 1-aminocyclopropane-1-carboxylicacid synthase; 8, AdoMet decarboxylase; 9, threonine synthase. Note that AVG inhibits both cystathionine g-synthase and 1-aminocyclopropane-1-carboxylic acid synthase.
(TIF)

S1 Table Silk viability information of different inbred lines and their corresponding hybrids. (XLSX)

S2 Table Peptide information for protein spots with significant changes based on MS analysis. (XLSX)

S3 Table Levels of differentially accumulated proteins in the inbred lines. (XLSX)

S4 Table Differentially accumulated protein spots in protein–protein interaction networks simulated by the String software, with COG functions. (XLSX)

## Abbreviations

CRPs: cysteine-rich peptides
Hcy: homocysteine
AdoMet: S-adenosylmethionine
MTA: methylthioadenosine
APT1: Adenine Phosphoribosyl Transferase 1
RuBisCO: Ribulose-1,5-bisphosphate carboxylase/oxygenase
ABP: Auxin-binding protein 1
AdoHcy: S-adenosylhomocysteine
Ade: adenine
MS: mass spectrometry
IEF: isoelectri focusing
MALDI-TOF: matrix-assisted laser desorption ionization-time of flight
PMF: peptide-map-fingerprinting
GO: Gene Ontology
KEGG: Kyoto Encyclopedia of Genes and Genomes
COG: Clusters of Orthologous Groups
KOGs: eukaryotic orthologous groups
LSD: least-significant difference
