# Supplementary material for: Proteomic Analysis of Silk Viability in Maize Inbred Lines and Their Corresponding Hybrids
Source: PLoS One. 2015 Dec 2;10(12):e0144050. doi: 10.1371/journal.pone.0144050 (PMC4668103; doi:10.1371/journal.pone.0144050)
Supplement: S1 Fig — (DOCX) [file pone.0144050.s001.docx]

**A**

Xun928 Xun928

4 PI 7

4 PI 7

100KD MW 14KD


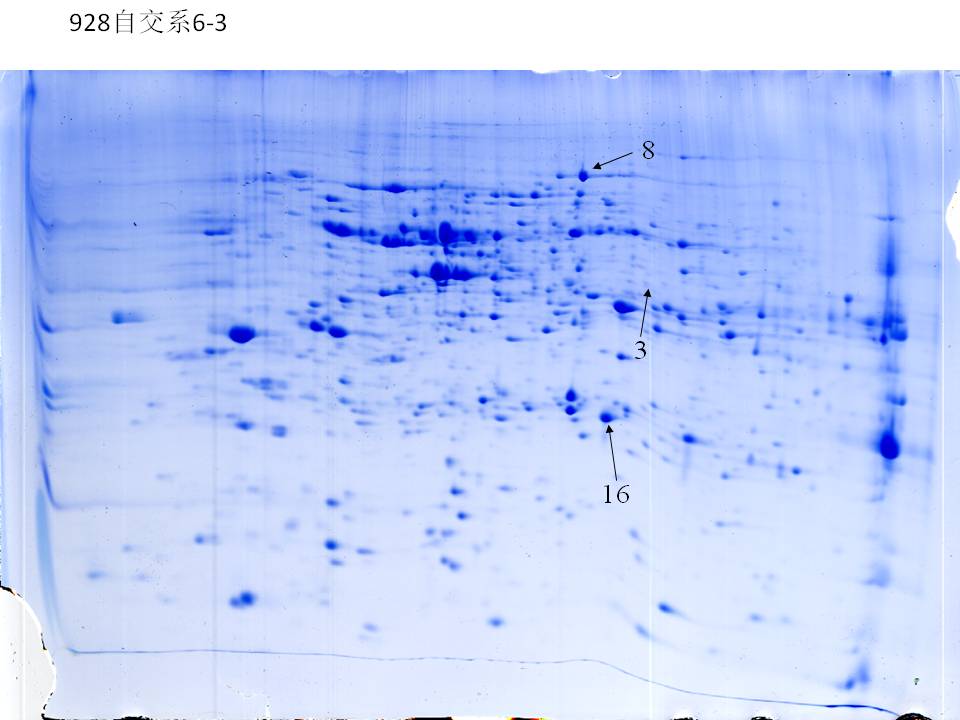

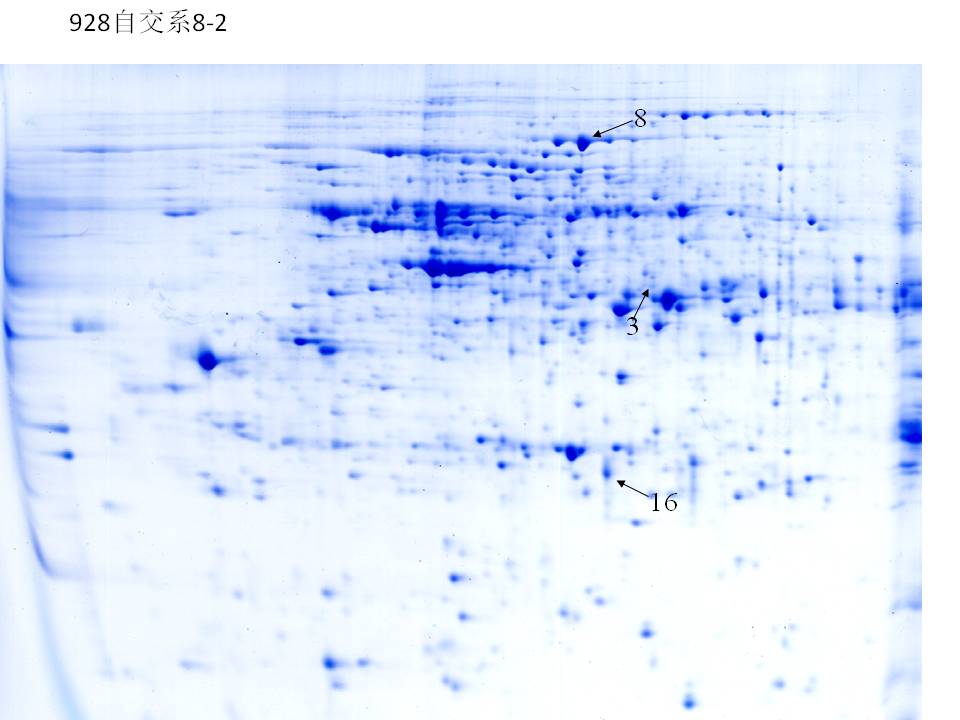


D_8_

D_6_

100KD MW 14KD

Xun928 Xun928

4 PI 7

100KD MW 14KD

4 PI 7


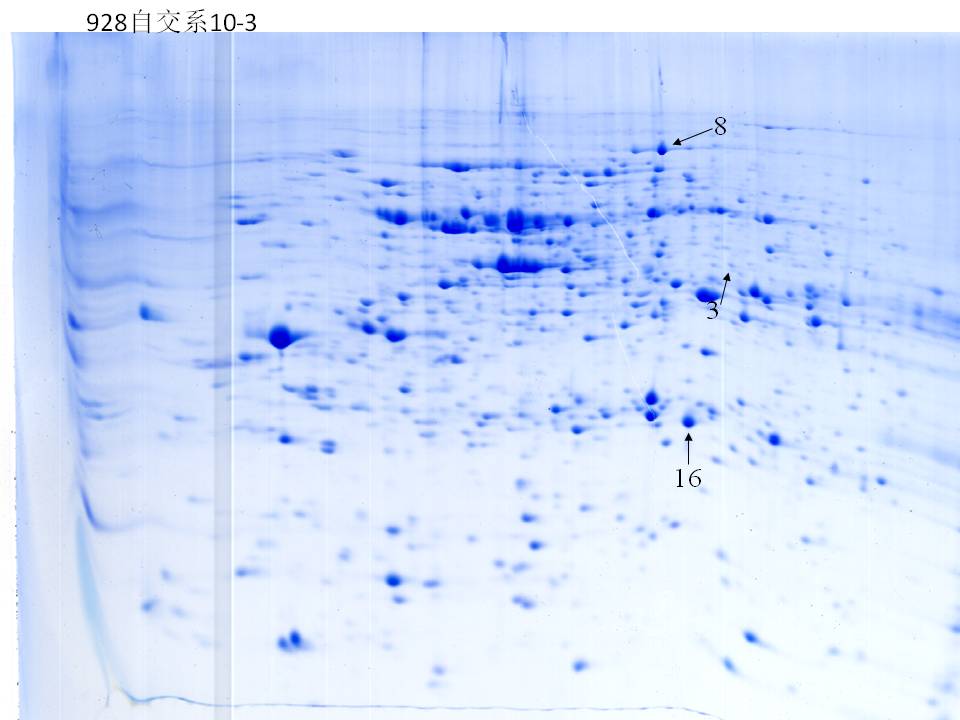

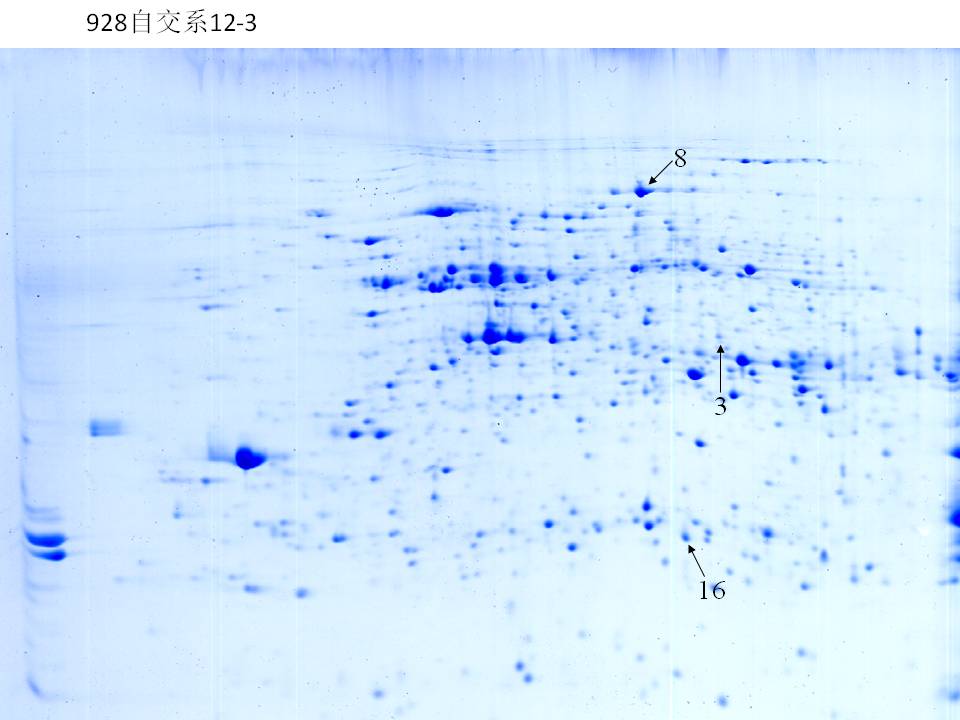


D_12_

D_10_

100KD MW 14KD

**B**

4 PI 7

Lx9801 Lx9801

4 PI 7


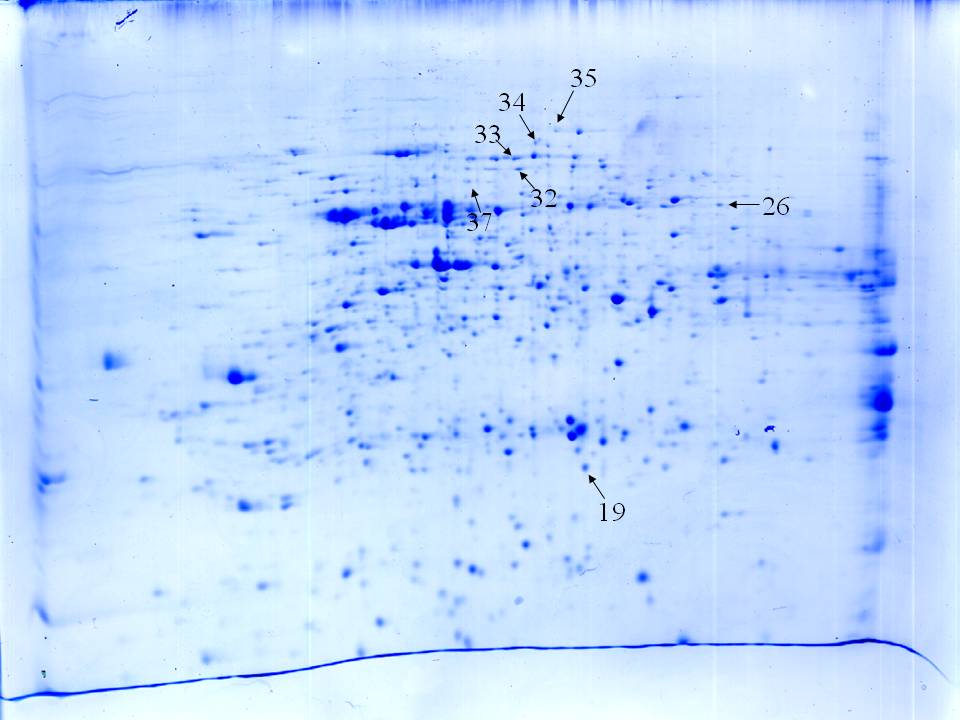

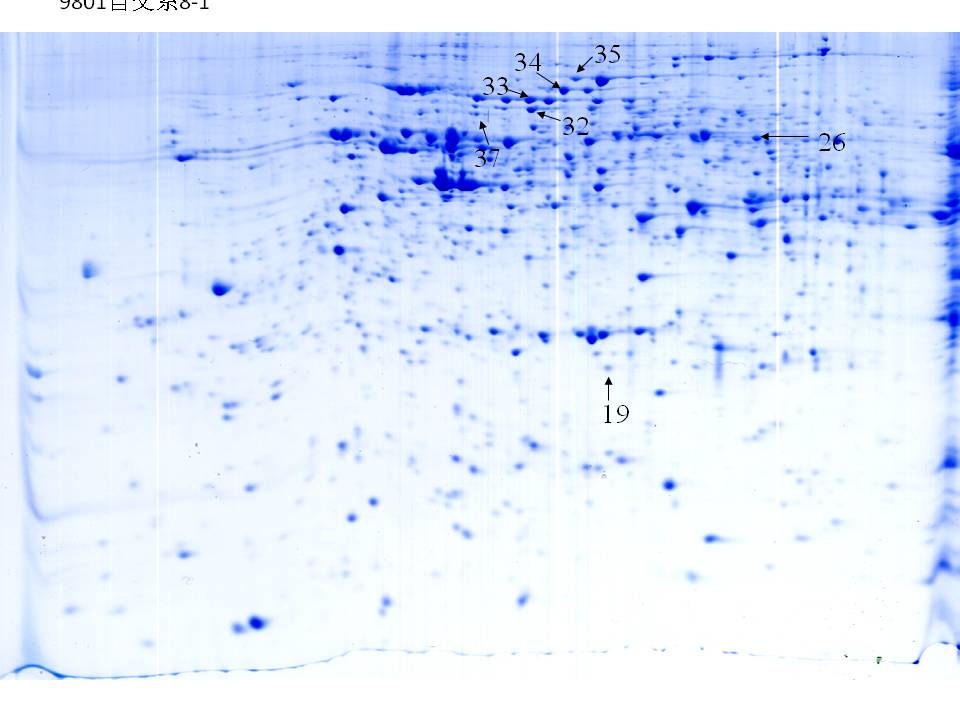


D_8_

D_10_

D_6_

100KD MW 14KD

100KD MW 14KD

Lx9801 Lx9801

4 PI 7

4 PI 7


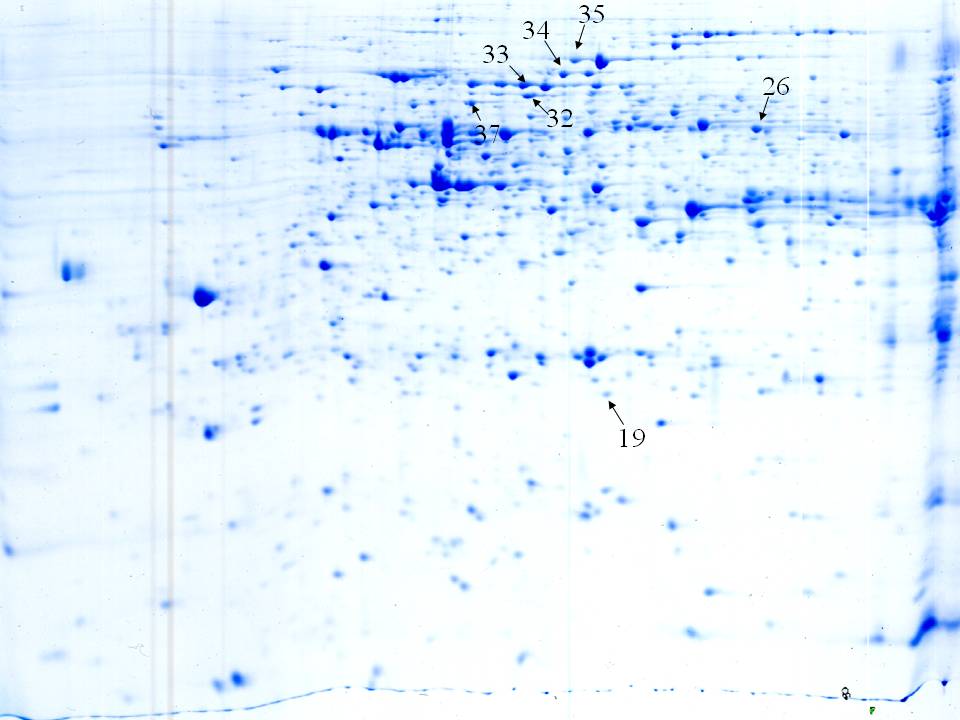

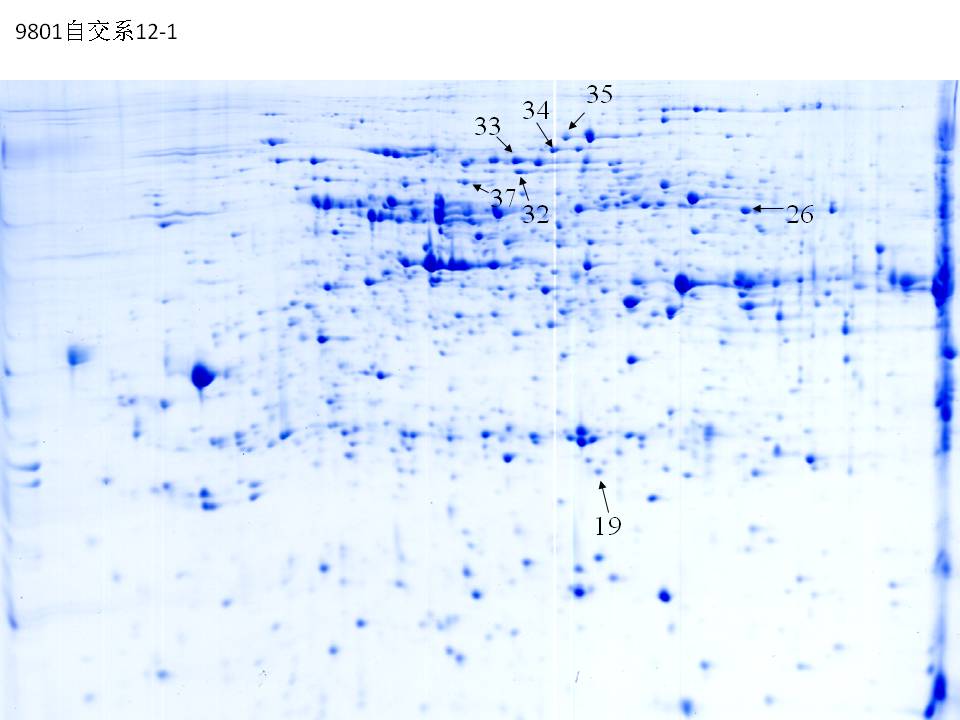


D_12_

100KD MW 14KD

100KD MW 14KD

**C**

Zong3 Zong3

4 PI 7

4 PI 7


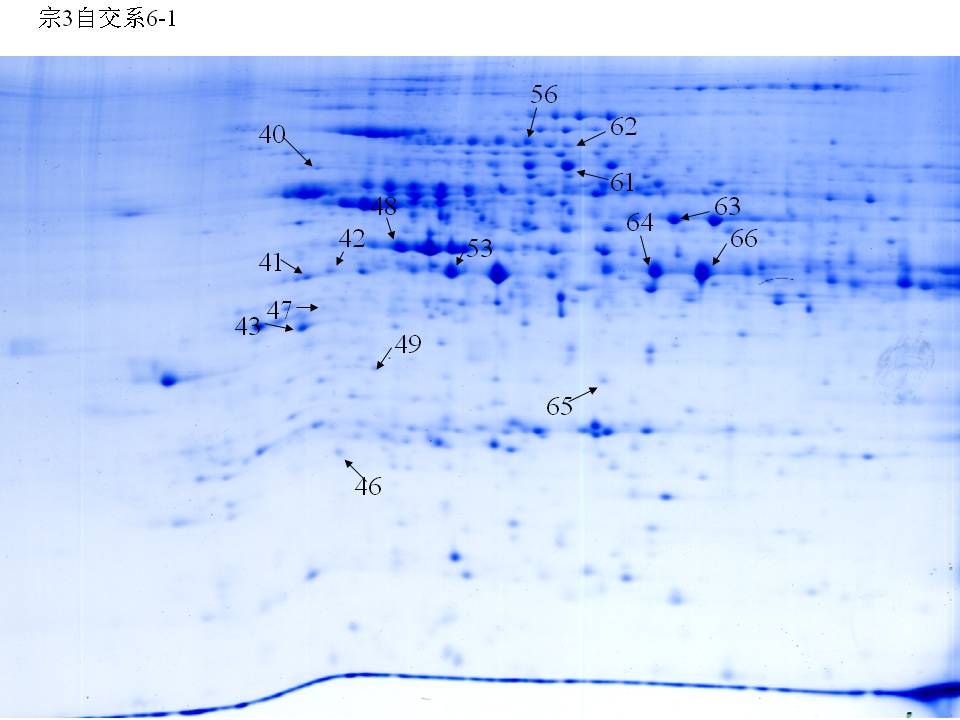

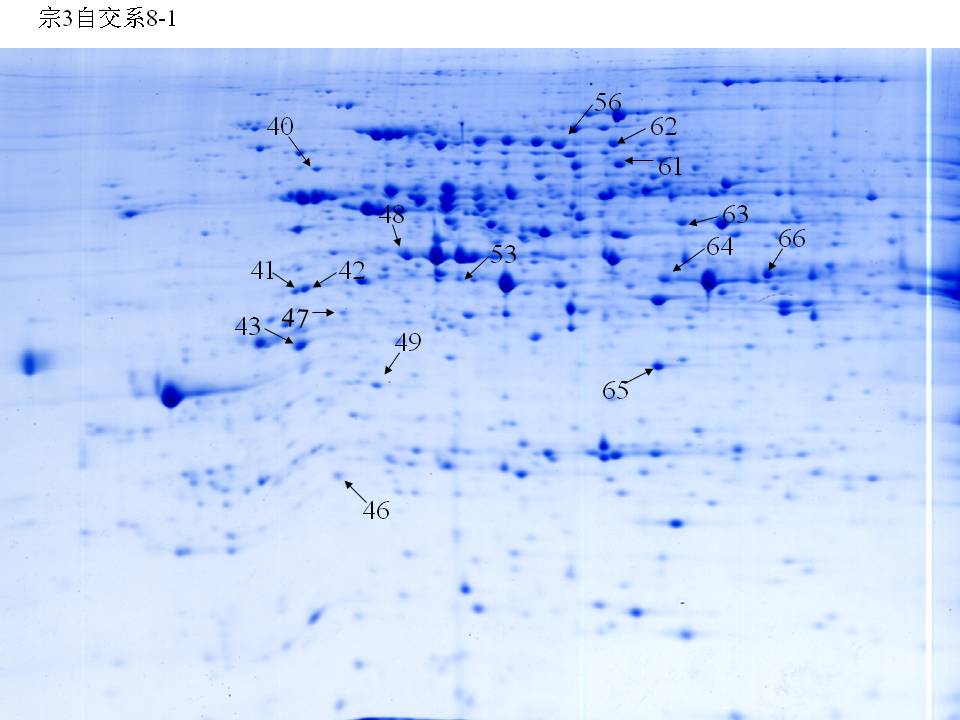


D_8_

D_6_

D_10_

100KD MW 14KD

100KD MW 14KD

4 PI 7

4 PI 7

Zong3 Zong3

100KD MW 14KD


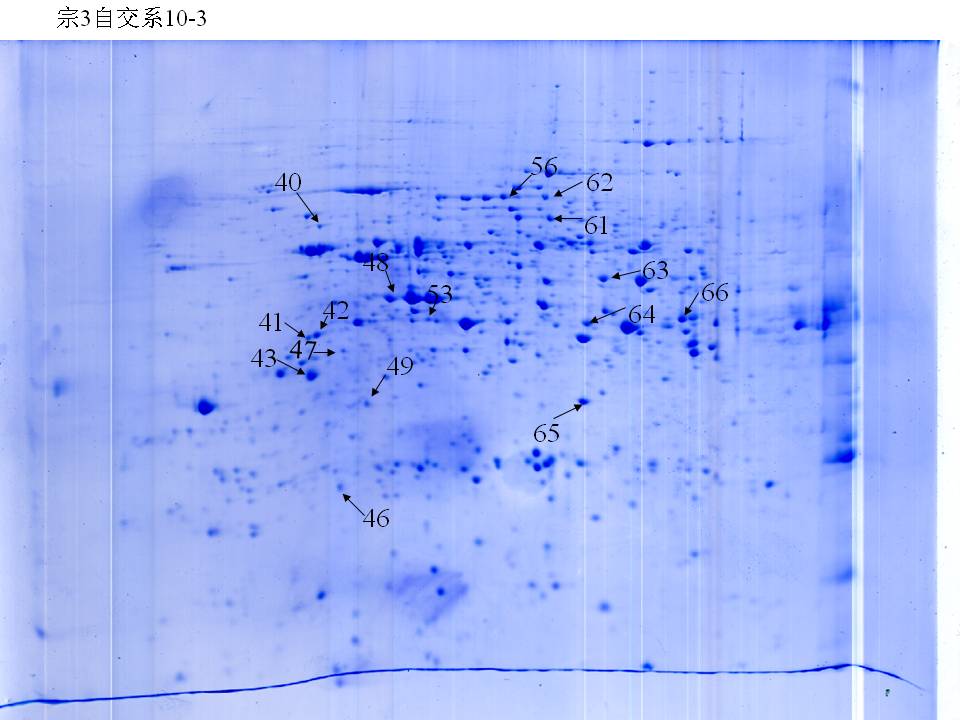

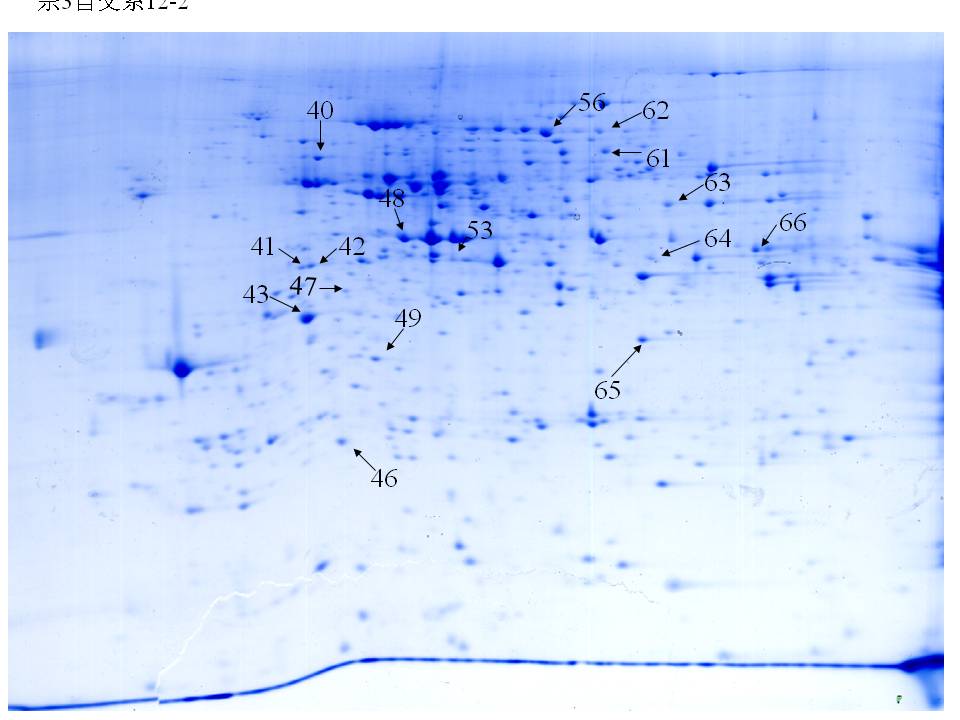


D_12_

100KD MW 14KD
