# Supplementary material for: Proteomic Analysis of Silk Viability in Maize Inbred Lines and Their Corresponding Hybrids
Source: PLoS One. 2015 Dec 2;10(12):e0144050. doi: 10.1371/journal.pone.0144050 (PMC4668103; doi:10.1371/journal.pone.0144050)
Supplement: S2 Fig — (DOCX) [file pone.0144050.s002.docx]

**A**

Xun928 Xun928×Zong3 Zong3

4 PI 7

100KD MW 14KD

4 PI 7

100KD MW 14KD

4 PI 7

100KD MW 14KD


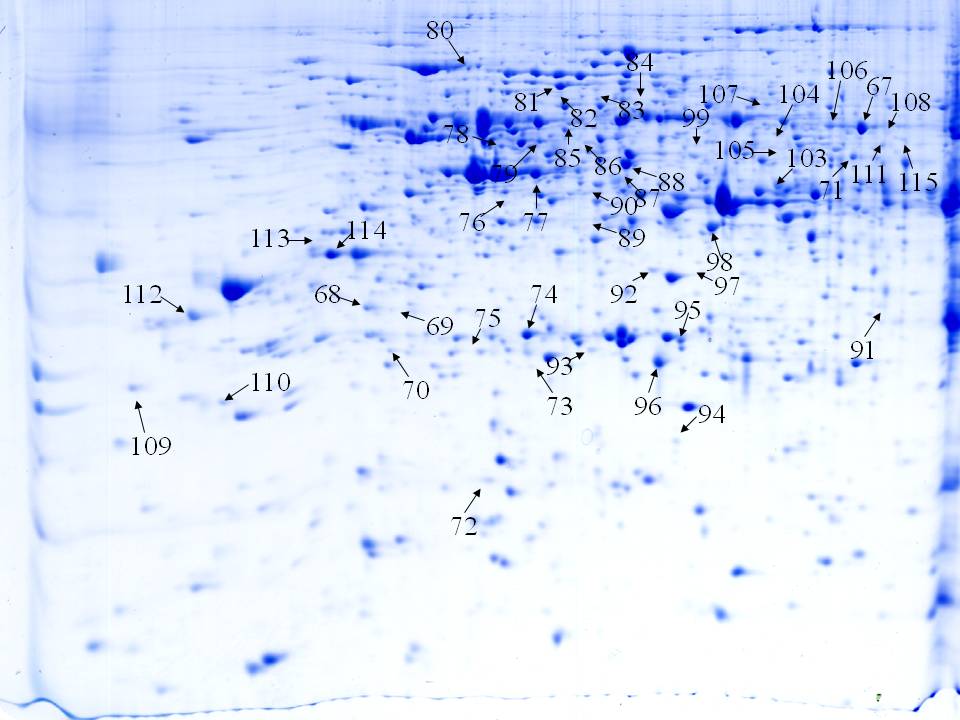

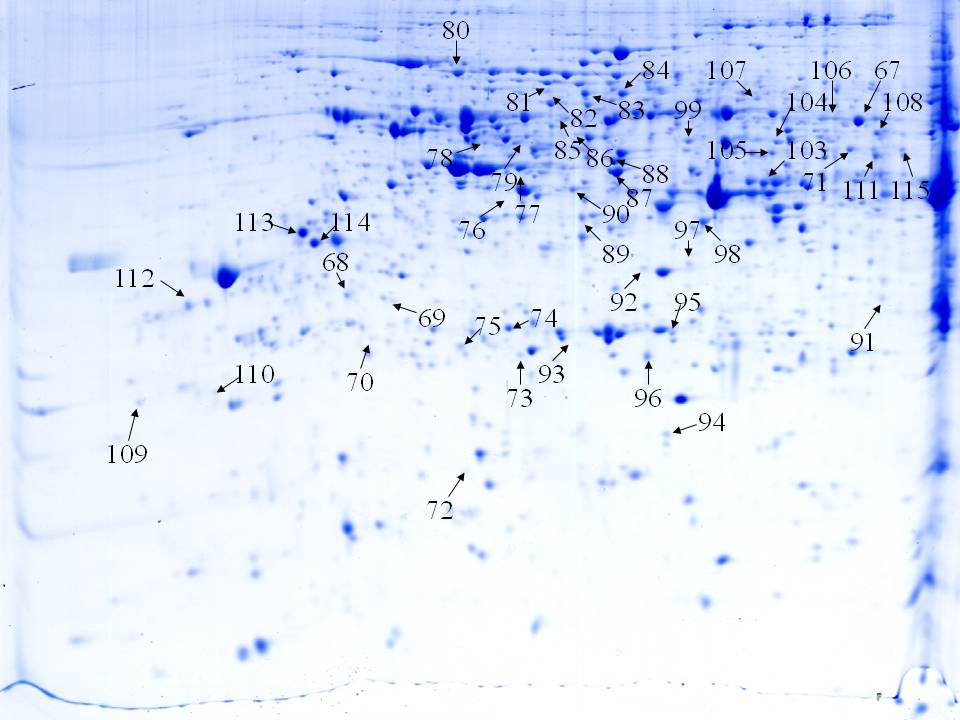

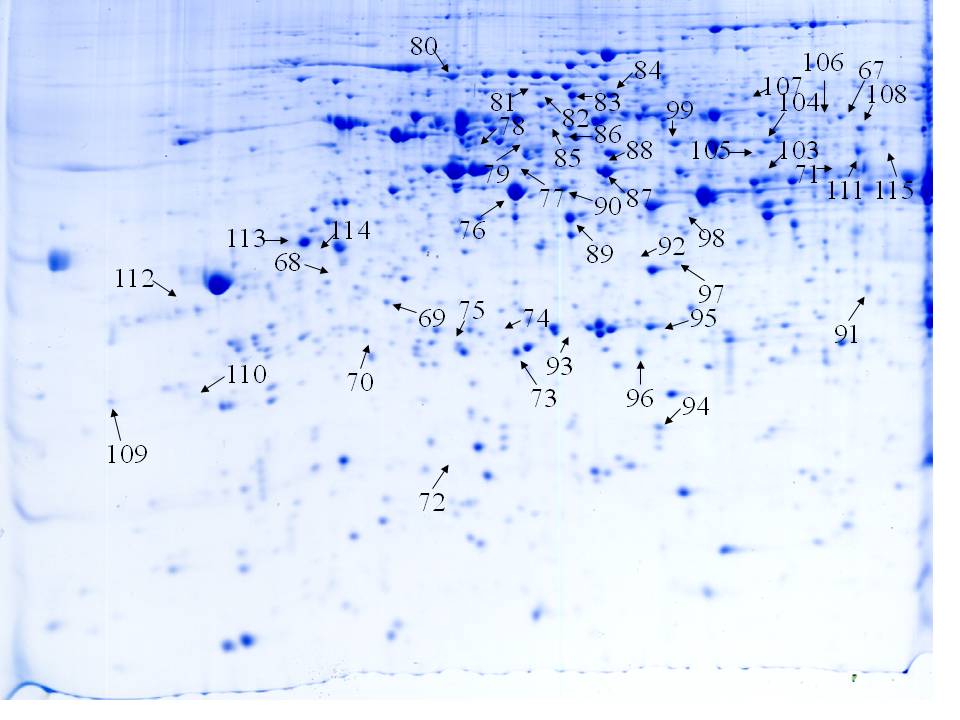


D_8_

**B**

100KD MW 14KD

4 PI 7

Xun928 Xun928×Zong3 Zong3

100KD MW 14KD

4 PI 7

100KD MW 14KD

4 PI 7


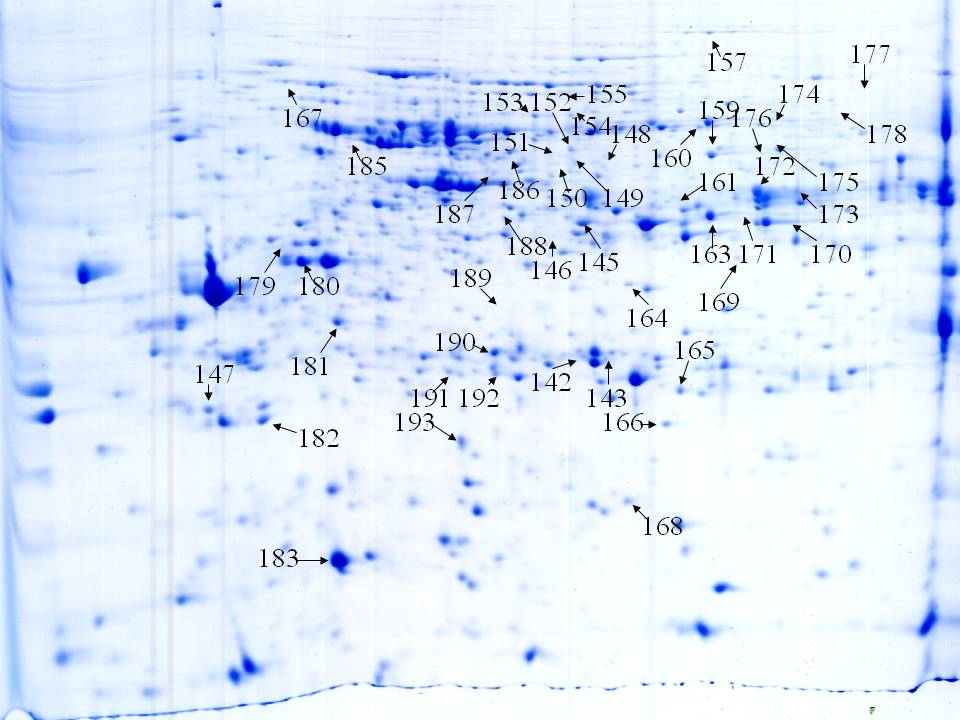

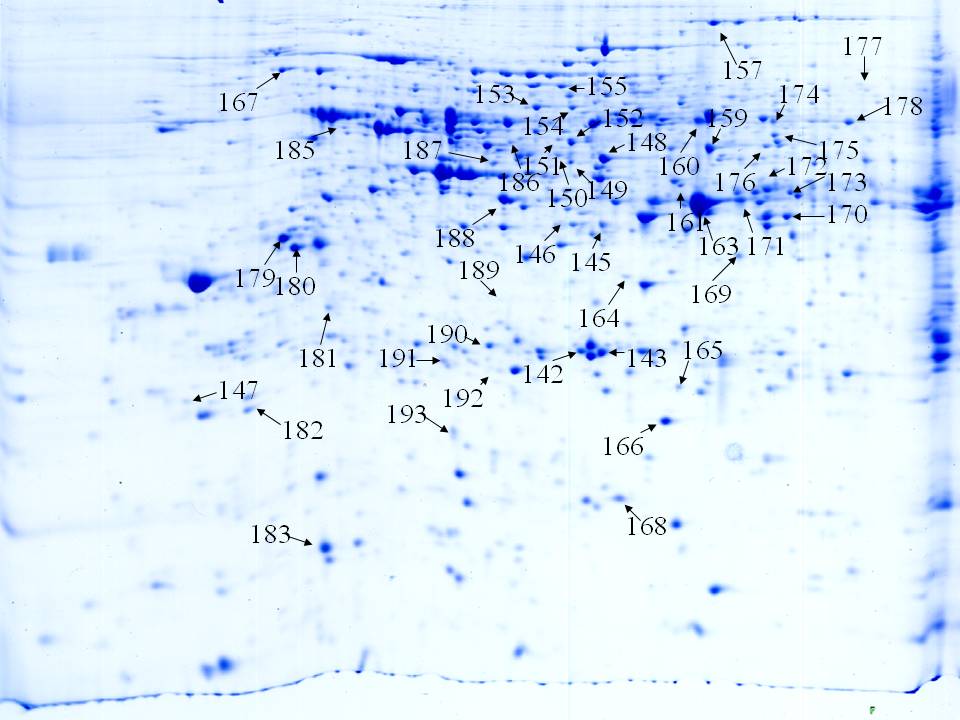

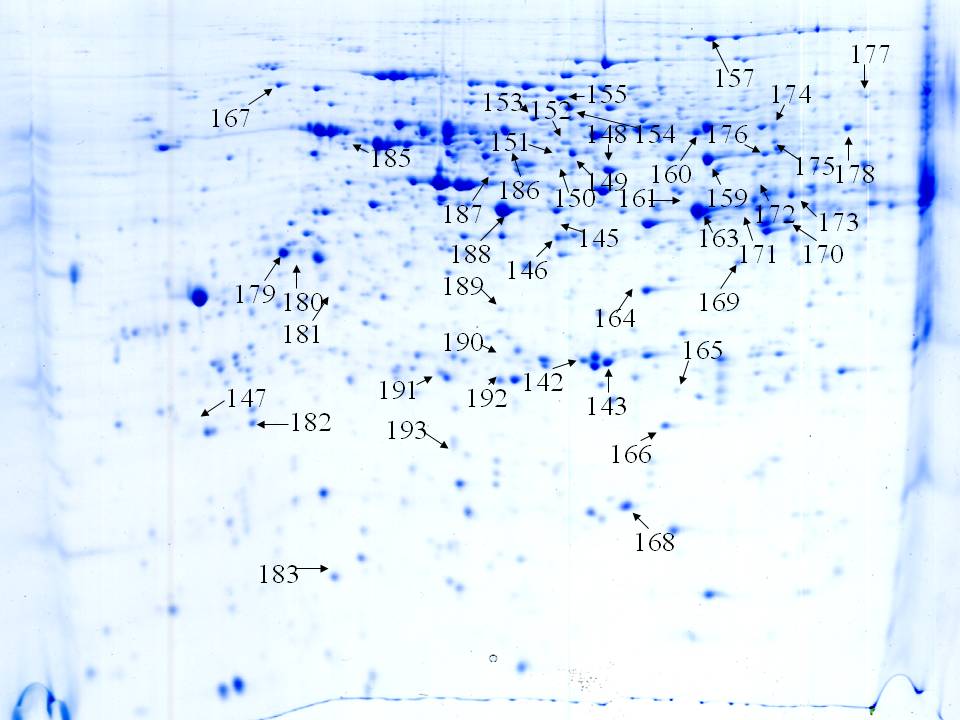


D_10_

**C**

Xun928 Xun928×Zong3 Zong3

100KD MW 14KD

4 PI 7

100KD MW 14KD

4 PI 7

100KD MW 14KD

4 PI 7


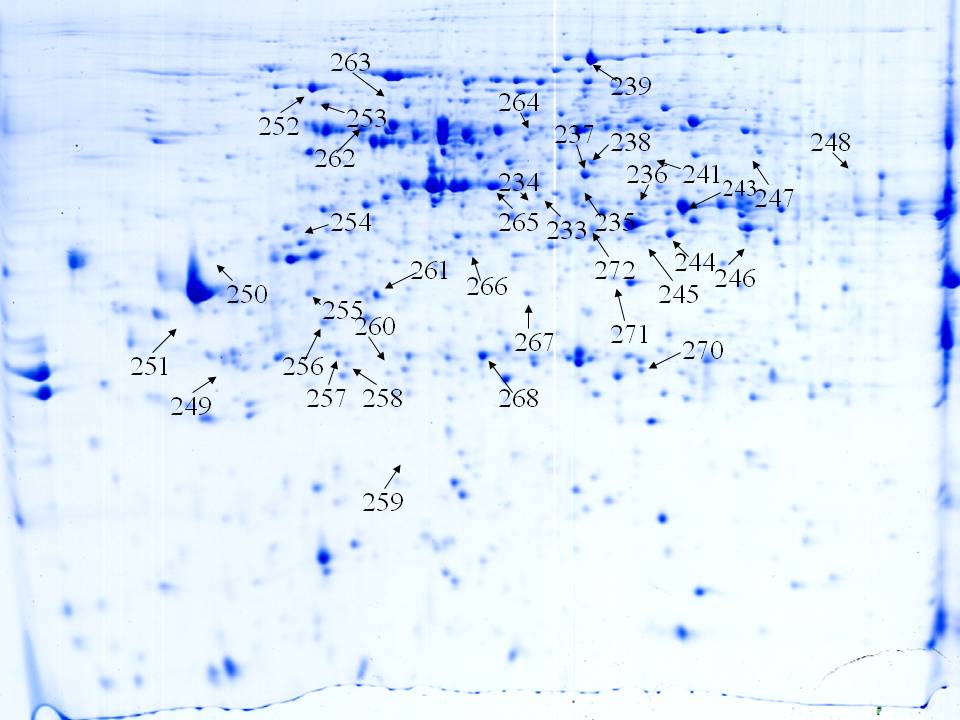

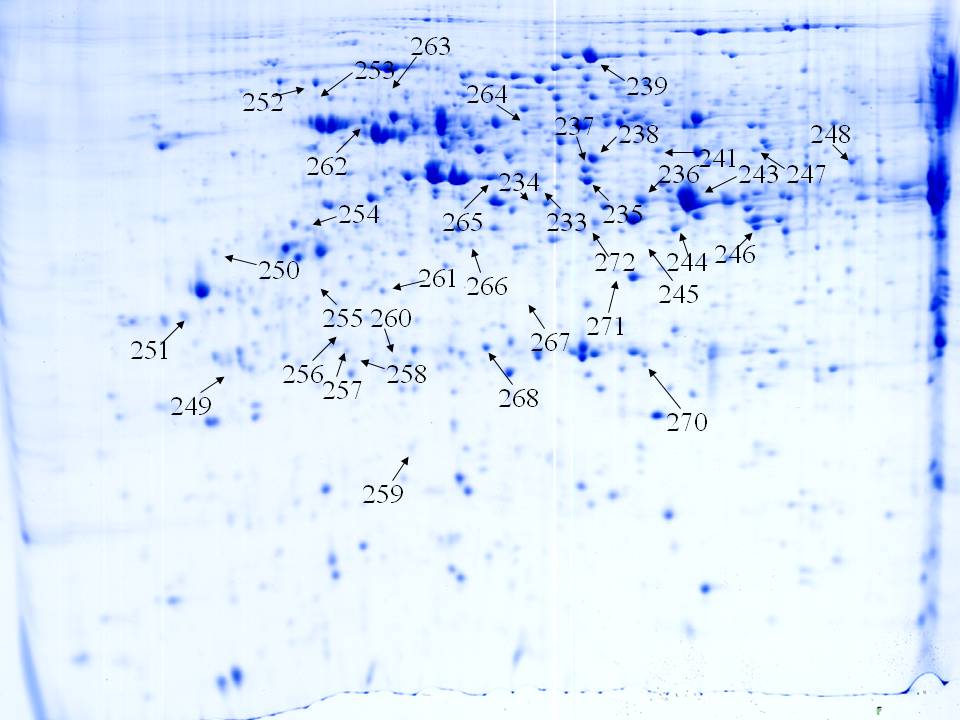

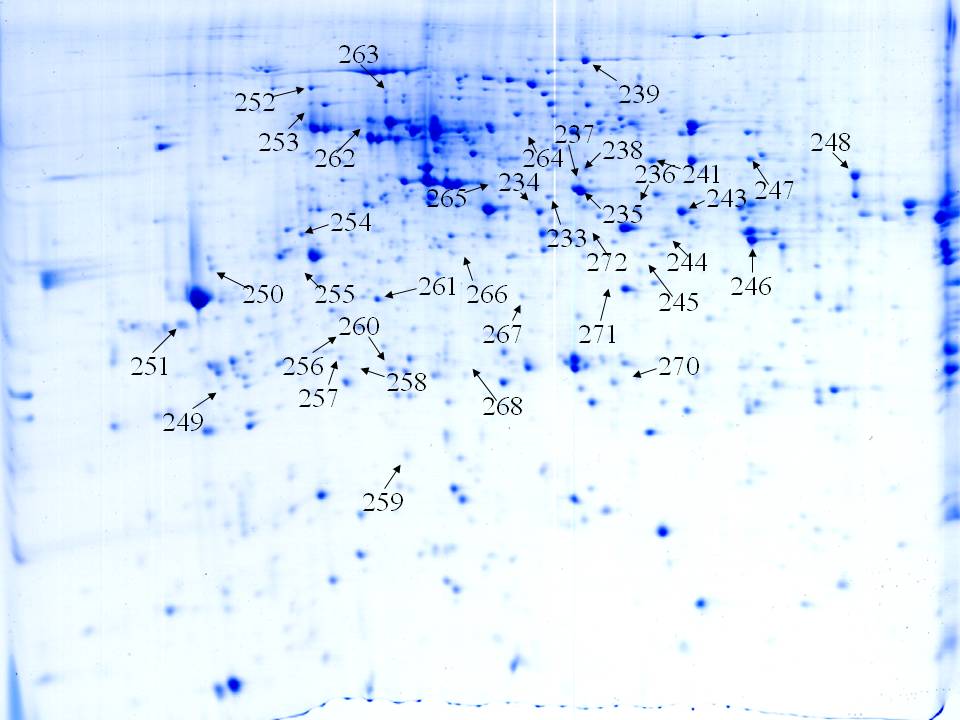


D_12_

**D**

Lx9801 Lx9801×Zong3 Zong3

100KD MW 14KD

4 PI 7

100KD MW 14KD

4 PI 7

100KD MW 14KD

4 PI 7


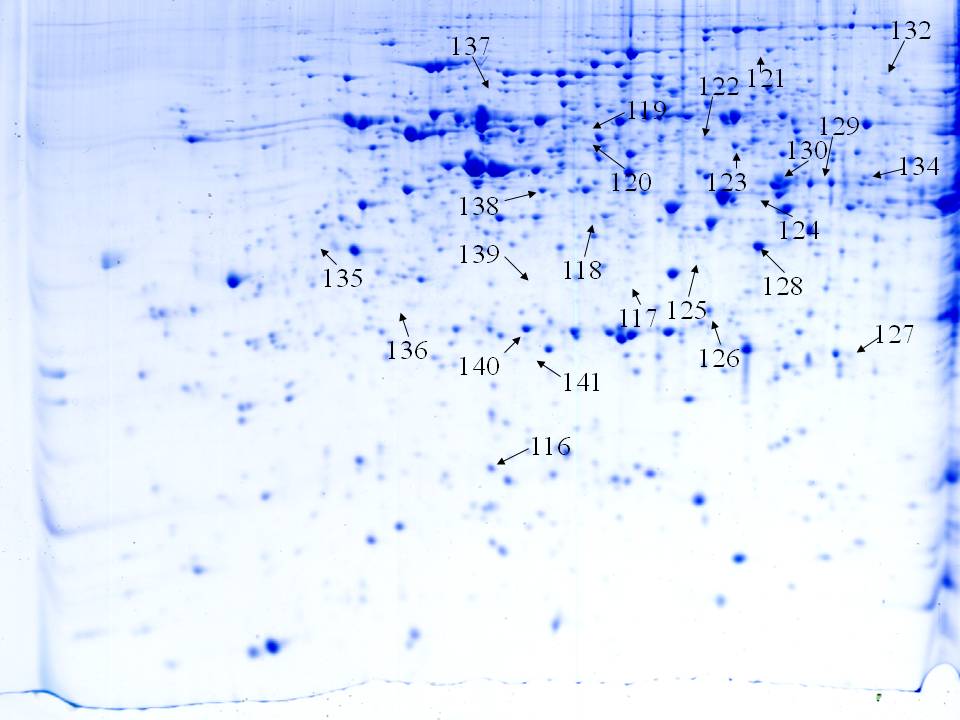

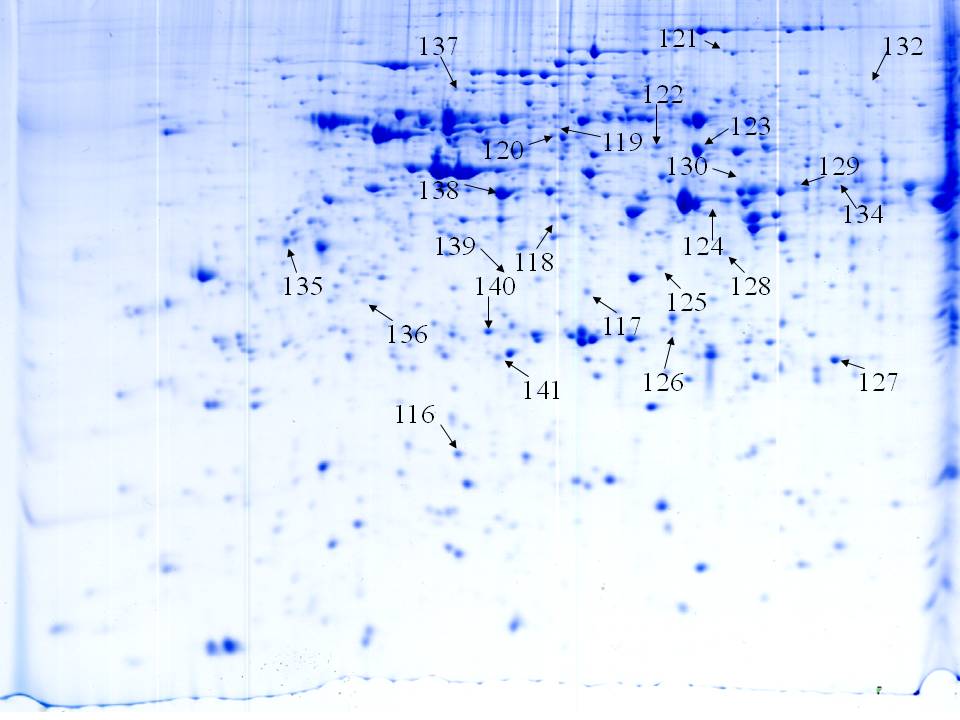

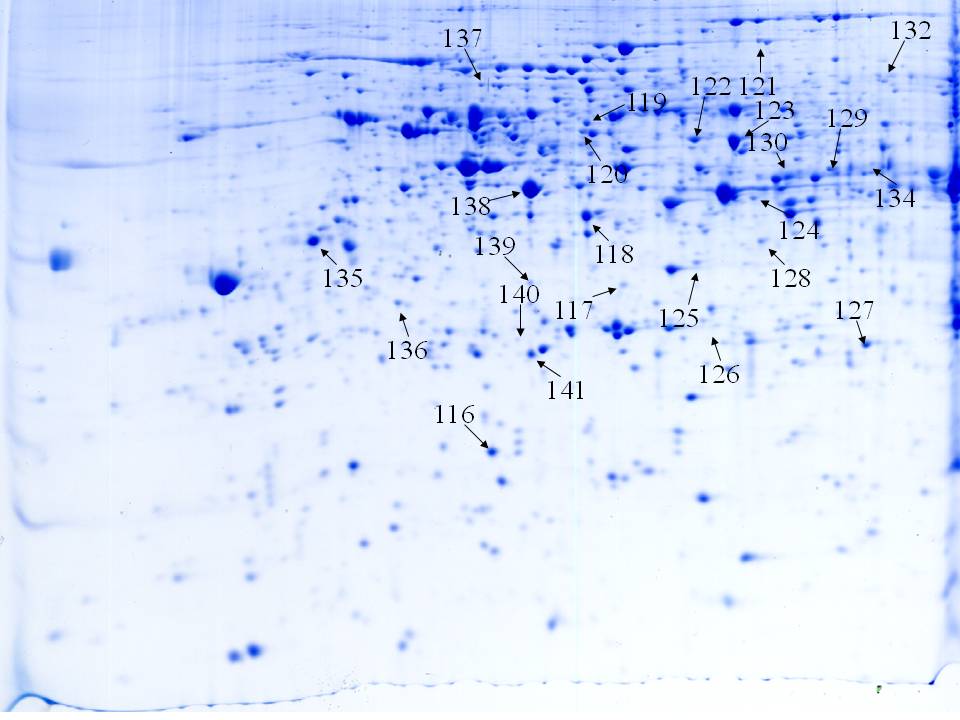


D_8_

**E**

Lx9801 Lx9801×Zong3 Zong3

100KD MW 14KD

4 PI 7

4 PI 7

100KD MW 14KD

4 PI 7

100KD MW 14KD


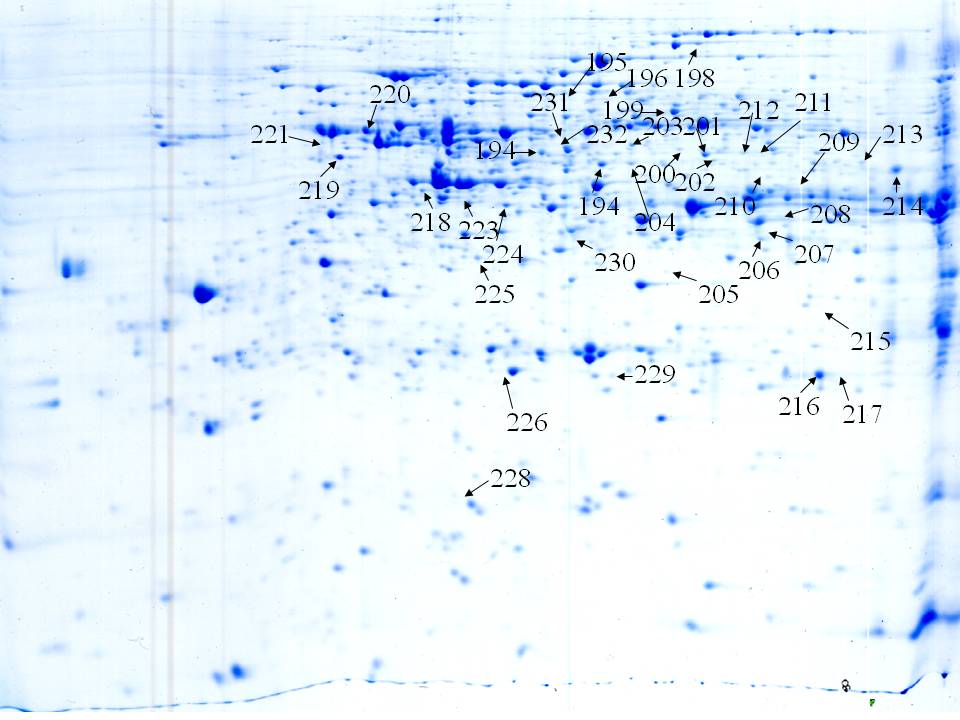

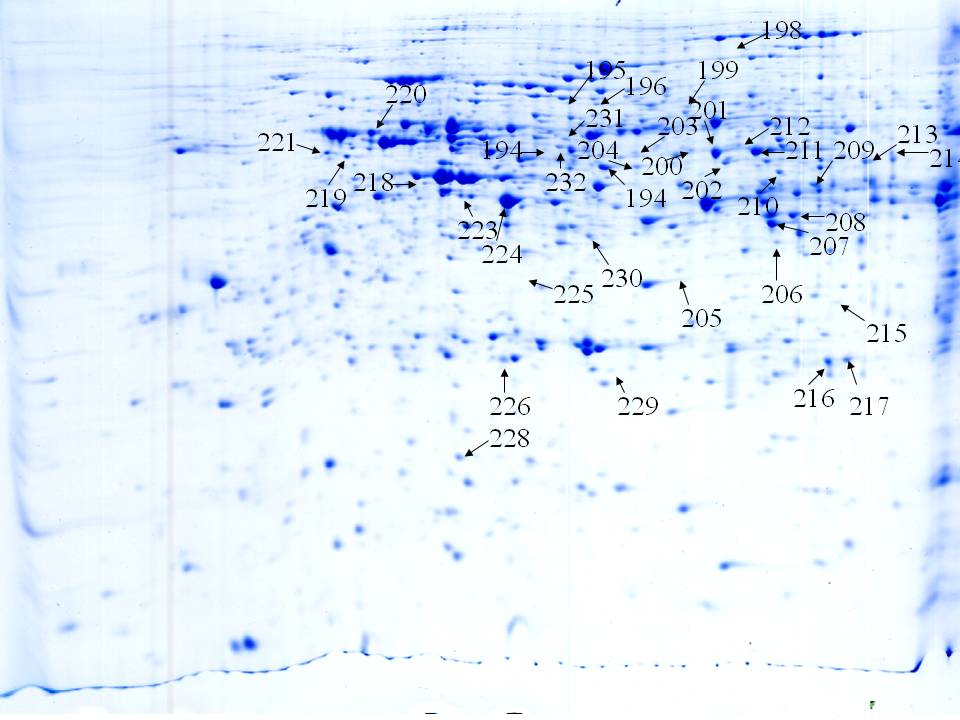

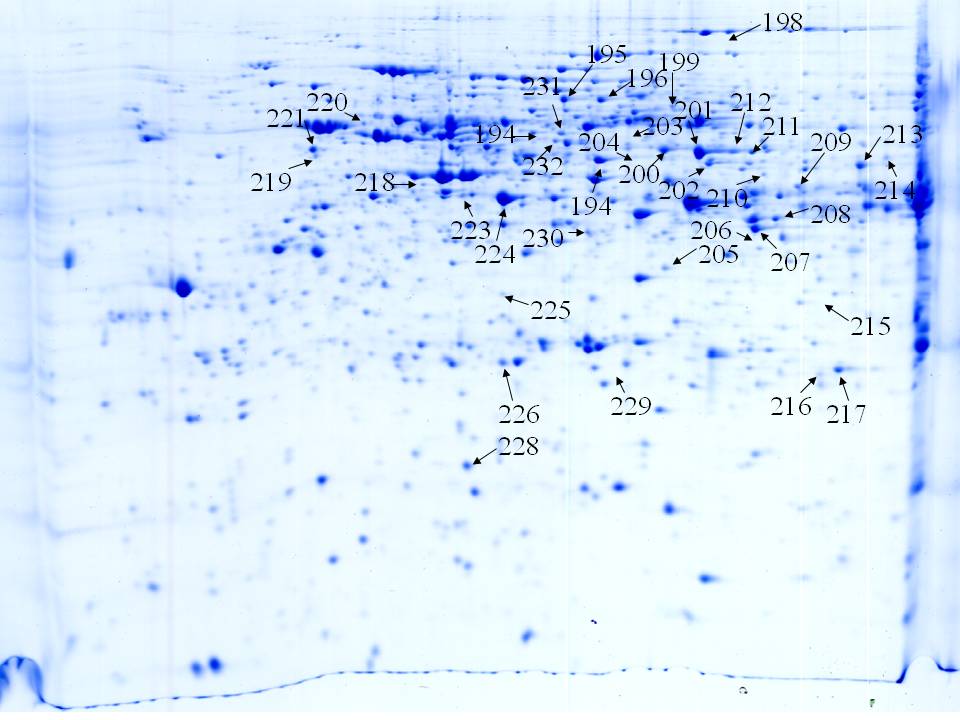


D_10_

**F**

4 PI 7

100KD MW 14KD

Lx9801 Lx9801×Zong3 Zong3

4 PI 7

100KD MW 14KD

100KD MW 14KD

4 PI 7


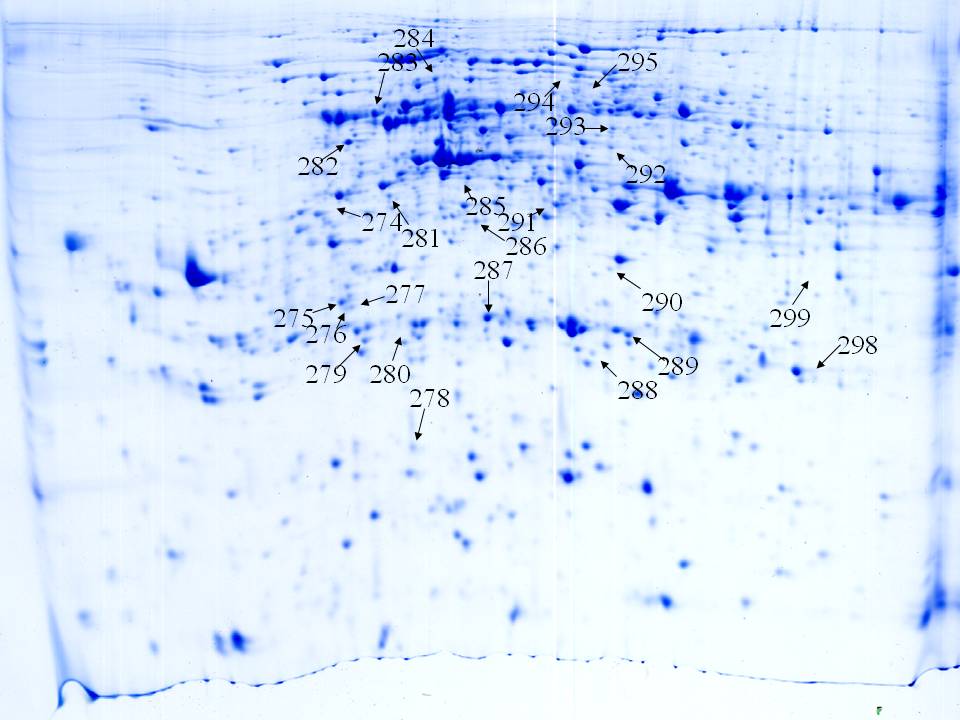

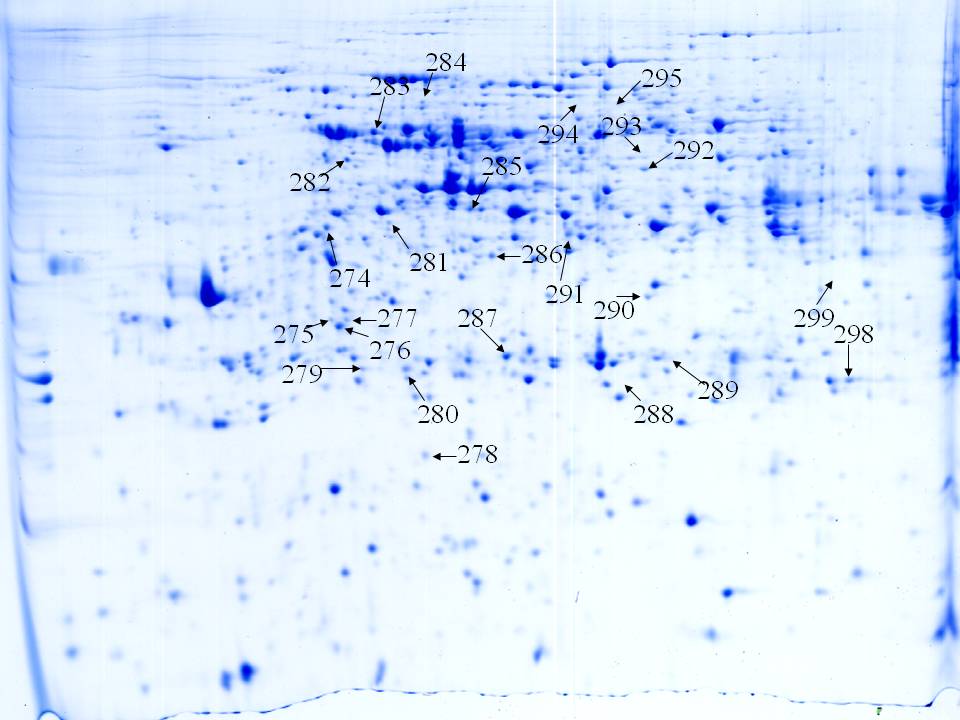

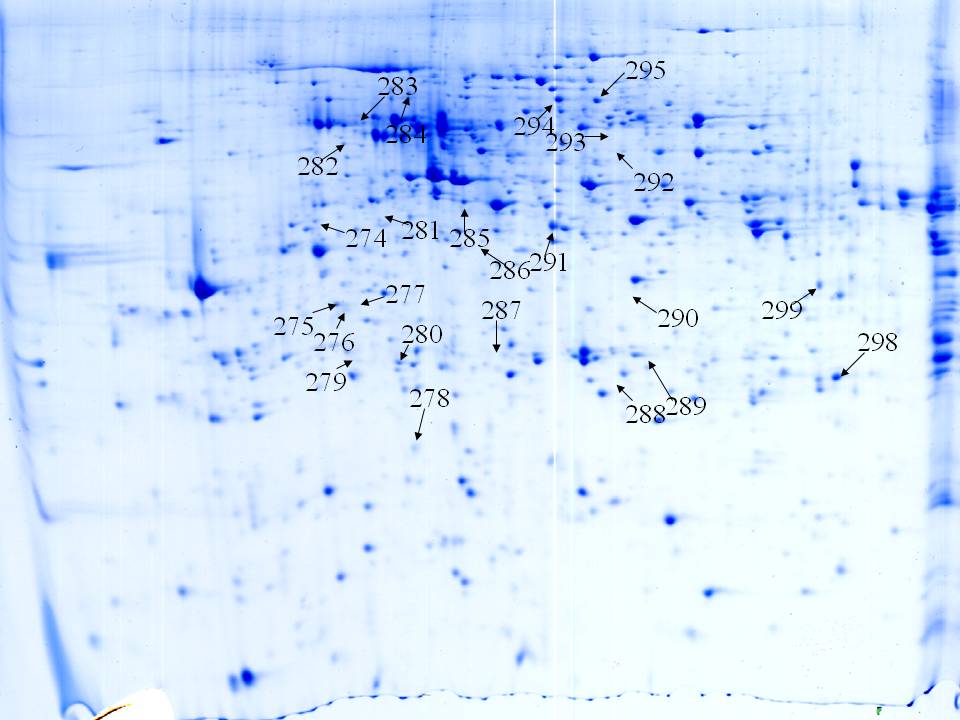


D_12_
