# Supplementary figures and images for: Proteomic Analysis of Silk Viability in Maize Inbred Lines and Their Corresponding Hybrids
Source: PLoS One. 2015 Dec 2;10(12):e0144050. doi: 10.1371/journal.pone.0144050 (PMC4668103; doi:10.1371/journal.pone.0144050)

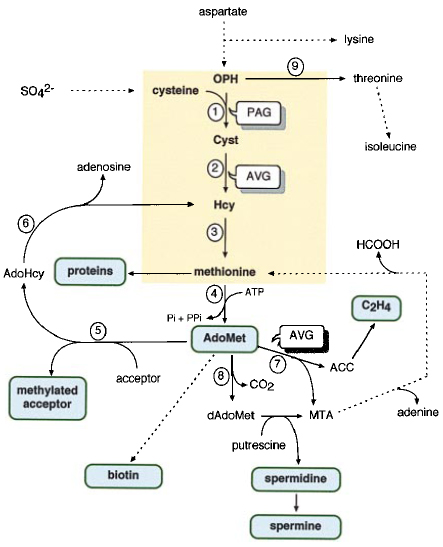

Supplement: S3 Fig — Enzymes: 1, cystathionine g-synthase; 2, cystathionine b-lyase; 3, methionine synthase; 4, AdoMet synthetase; 5, AdoMet-dependent methylase; 6, AdoHcy hydrolase; 7, 1-aminocyclopropane-1-carboxylicacid synthase; 8, AdoMet decarboxylase; 9, threonine synthase. Note that AVG inhibits both cystathionine g-synthase and 1-aminocyclopropane-1-carboxylic acid synthase. (TIF) [file pone.0144050.s003.tif]
